# Search for heavy resonances decaying to two Higgs bosons in final states containing four b quarks

**DOI:** 10.1140/epjc/s10052-016-4206-6

**Published:** 2016-07-04

**Authors:** V. Khachatryan, A. M. Sirunyan, A. Tumasyan, W. Adam, E. Asilar, T. Bergauer, J. Brandstetter, E. Brondolin, M. Dragicevic, J. Erö, M. Flechl, M. Friedl, R. Frühwirth, V. M. Ghete, C. Hartl, N. Hörmann, J. Hrubec, M. Jeitler, A. König, M. Krammer, I. Krätschmer, D. Liko, T. Matsushita, I. Mikulec, D. Rabady, N. Rad, B. Rahbaran, H. Rohringer, J. Schieck, R. Schöfbeck, J. Strauss, W. Treberer-Treberspurg, W. Waltenberger, C.-E. Wulz, V. Mossolov, N. Shumeiko, J. Suarez Gonzalez, S. Alderweireldt, T. Cornelis, E. A. De Wolf, X. Janssen, A. Knutsson, J. Lauwers, S. Luyckx, M. Van De Klundert, H. Van Haevermaet, P. Van Mechelen, N. Van Remortel, A. Van Spilbeeck, S. Abu Zeid, F. Blekman, J. D’Hondt, N. Daci, I. De Bruyn, K. Deroover, N. Heracleous, J. Keaveney, S. Lowette, S Moortgat, L. Moreels, A. Olbrechts, Q. Python, D. Strom, S. Tavernier, W. Van Doninck, P. Van Mulders, G. P. Van Onsem, I. Van Parijs, P. Barria, H. Brun, C. Caillol, B. Clerbaux, G. De Lentdecker, G. Fasanella, L. Favart, R. Goldouzian, A. Grebenyuk, G. Karapostoli, T. Lenzi, A. Léonard, T. Maerschalk, A. Marinov, L. Perniè, A. Randle-conde, T. Seva, C. Vander Velde, P. Vanlaer, R. Yonamine, F. Zenoni, F. Zhang, K. Beernaert, L. Benucci, A. Cimmino, S. Crucy, D. Dobur, A. Fagot, G. Garcia, M. Gul, J. Mccartin, A. A. Ocampo Rios, D. Poyraz, D. Ryckbosch, S. Salva, M. Sigamani, M. Tytgat, W. Van Driessche, E. Yazgan, N. Zaganidis, S. Basegmez, C. Beluffi, O. Bondu, S. Brochet, G. Bruno, A. Caudron, L. Ceard, S. De Visscher, C. Delaere, M. Delcourt, D. Favart, L. Forthomme, A. Giammanco, A. Jafari, P. Jez, M. Komm, V. Lemaitre, A. Mertens, M. Musich, C. Nuttens, L. Perrini, K. Piotrzkowski, A. Popov, L. Quertenmont, M. Selvaggi, M. Vidal Marono, N. Beliy, G. H. Hammad, W. L. Aldá Júnior, F. L. Alves, G. A. Alves, L. Brito, M. Correa Martins Junior, M. Hamer, C. Hensel, A. Moraes, M. E. Pol, P. Rebello Teles, E. Belchior Batista Das Chagas, W. Carvalho, J. Chinellato, A. Custódio, E. M. Da Costa, D. De Jesus Damiao, C. De Oliveira Martins, S. Fonseca De Souza, L. M. Huertas Guativa, H. Malbouisson, D. Matos Figueiredo, C. Mora Herrera, L. Mundim, H. Nogima, W. L. Prado Da Silva, A. Santoro, A. Sznajder, E. J. Tonelli Manganote, A. Vilela Pereira, S. Ahuja, C. A. Bernardes, A. De Souza Santos, S. Dogra, T. R. Fernandez Perez Tomei, E. M. Gregores, P. G. Mercadante, C. S. Moon, S. F. Novaes, Sandra S. Padula, D. Romero Abad, J. C. Ruiz Vargas, A. Aleksandrov, R. Hadjiiska, P. Iaydjiev, M. Rodozov, S. Stoykova, G. Sultanov, M. Vutova, A. Dimitrov, I. Glushkov, L. Litov, B. Pavlov, P. Petkov, W. Fang, M. Ahmad, J. G. Bian, G. M. Chen, H. S. Chen, M. Chen, T. Cheng, R. Du, C. H. Jiang, D. Leggat, R. Plestina, F. Romeo, S. M. Shaheen, A. Spiezia, J. Tao, C. Wang, Z. Wang, H. Zhang, C. Asawatangtrakuldee, Y. Ban, Q. Li, S. Liu, Y. Mao, S. J. Qian, D. Wang, Z. Xu, C. Avila, A. Cabrera, L. F. Chaparro Sierra, C. Florez, J. P. Gomez, B. Gomez Moreno, J. C. Sanabria, N. Godinovic, D. Lelas, I. Puljak, P. M. Ribeiro Cipriano, Z. Antunovic, M. Kovac, V. Brigljevic, K. Kadija, J. Luetic, S. Micanovic, L. Sudic, A. Attikis, G. Mavromanolakis, J. Mousa, C. Nicolaou, F. Ptochos, P. A. Razis, H. Rykaczewski, M. Finger, M. Finger, Y. Assran, A. Ellithi Kamel, A. Mahrous, A. Radi, B. Calpas, M. Kadastik, M. Murumaa, M. Raidal, A. Tiko, C. Veelken, P. Eerola, J. Pekkanen, M. Voutilainen, J. Härkönen, V. Karimäki, R. Kinnunen, T. Lampén, K. Lassila-Perini, S. Lehti, T. Lindén, P. Luukka, T. Peltola, J. Tuominiemi, E. Tuovinen, L. Wendland, J. Talvitie, T. Tuuva, M. Besancon, F. Couderc, M. Dejardin, D. Denegri, B. Fabbro, J. L. Faure, C. Favaro, F. Ferri, S. Ganjour, A. Givernaud, P. Gras, G. Hamel de Monchenault, P. Jarry, E. Locci, M. Machet, J. Malcles, J. Rander, A. Rosowsky, M. Titov, A. Zghiche, A. Abdulsalam, I. Antropov, S. Baffioni, F. Beaudette, P. Busson, L. Cadamuro, E. Chapon, C. Charlot, O. Davignon, N. Filipovic, R. Granier de Cassagnac, M. Jo, S. Lisniak, L. Mastrolorenzo, P. Miné, I. N. Naranjo, M. Nguyen, C. Ochando, G. Ortona, P. Paganini, P. Pigard, S. Regnard, R. Salerno, Y. Sirois, T. Strebler, Y. Yilmaz, A. Zabi, J.-L. Agram, J. Andrea, A. Aubin, D. Bloch, J.-M. Brom, M. Buttignol, E. C. Chabert, N. Chanon, C. Collard, E. Conte, X. Coubez, J.-C. Fontaine, D. Gelé, U. Goerlach, C. Goetzmann, A.-C. Le Bihan, J. A. Merlin, K. Skovpen, P. Van Hove, S. Gadrat, S. Beauceron, C. Bernet, G. Boudoul, E. Bouvier, C. A. Carrillo Montoya, R. Chierici, D. Contardo, B. Courbon, P. Depasse, H. El Mamouni, J. Fan, J. Fay, S. Gascon, M. Gouzevitch, B. Ille, F. Lagarde, I. B. Laktineh, M. Lethuillier, L. Mirabito, A. L. Pequegnot, S. Perries, J. D. Ruiz Alvarez, D. Sabes, V. Sordini, M. Vander Donckt, P. Verdier, S. Viret, T. Toriashvili, L. Rurua, C. Autermann, S. Beranek, L. Feld, A. Heister, M. K. Kiesel, K. Klein, M. Lipinski, A. Ostapchuk, M. Preuten, F. Raupach, S. Schael, J. F. Schulte, T. Verlage, H. Weber, V. Zhukov, M. Ata, M. Brodski, E. Dietz-Laursonn, D. Duchardt, M. Endres, M. Erdmann, S. Erdweg, T. Esch, R. Fischer, A. Güth, T. Hebbeker, C. Heidemann, K. Hoepfner, S. Knutzen, M. Merschmeyer, A. Meyer, P. Millet, S. Mukherjee, M. Olschewski, K. Padeken, P. Papacz, T. Pook, M. Radziej, H. Reithler, M. Rieger, F. Scheuch, L. Sonnenschein, D. Teyssier, S. Thüer, V. Cherepanov, Y. Erdogan, G. Flügge, H. Geenen, M. Geisler, F. Hoehle, B. Kargoll, T. Kress, A. Künsken, J. Lingemann, A. Nehrkorn, A. Nowack, I. M. Nugent, C. Pistone, O. Pooth, A. Stahl, M. Aldaya Martin, I. Asin, N. Bartosik, O. Behnke, U. Behrens, K. Borras, A. Burgmeier, A. Campbell, C. Contreras-Campana, F. Costanza, C. Diez Pardos, G. Dolinska, S. Dooling, T. Dorland, G. Eckerlin, D. Eckstein, T. Eichhorn, G. Flucke, E. Gallo, J. Garay Garcia, A. Geiser, A. Gizhko, P. Gunnellini, J. Hauk, M. Hempel, H. Jung, A. Kalogeropoulos, O. Karacheban, M. Kasemann, P. Katsas, J. Kieseler, C. Kleinwort, I. Korol, W. Lange, J. Leonard, K. Lipka, A. Lobanov, W. Lohmann, R. Mankel, I.-A. Melzer-Pellmann, A. B. Meyer, G. Mittag, J. Mnich, A. Mussgiller, S. Naumann-Emme, A. Nayak, E. Ntomari, H. Perrey, D. Pitzl, R. Placakyte, A. Raspereza, B. Roland, M. Ö. Sahin, P. Saxena, T. Schoerner-Sadenius, C. Seitz, S. Spannagel, N. Stefaniuk, K. D. Trippkewitz, R. Walsh, C. Wissing, V. Blobel, M. Centis Vignali, A. R. Draeger, T. Dreyer, J. Erfle, E. Garutti, K. Goebel, D. Gonzalez, M. Görner, J. Haller, M. Hoffmann, R. S. Höing, A. Junkes, R. Klanner, R. Kogler, N. Kovalchuk, T. Lapsien, T. Lenz, I. Marchesini, D. Marconi, M. Meyer, M. Niedziela, D. Nowatschin, J. Ott, F. Pantaleo, T. Peiffer, A. Perieanu, N. Pietsch, J. Poehlsen, C. Sander, C. Scharf, P. Schleper, E. Schlieckau, A. Schmidt, S. Schumann, J. Schwandt, V. Sola, H. Stadie, G. Steinbrück, F. M. Stober, H. Tholen, D. Troendle, E. Usai, L. Vanelderen, A. Vanhoefer, B. Vormwald, C. Barth, C. Baus, J. Berger, C. Böser, E. Butz, T. Chwalek, F. Colombo, W. De Boer, A. Descroix, A. Dierlamm, S. Fink, F. Frensch, R. Friese, M. Giffels, A. Gilbert, D. Haitz, F. Hartmann, S. M. Heindl, U. Husemann, I. Katkov, A. Kornmayer, P. Lobelle Pardo, B. Maier, H. Mildner, M. U. Mozer, T. Müller, Th. Müller, M. Plagge, G. Quast, K. Rabbertz, S. Röcker, F. Roscher, M. Schröder, G. Sieber, H. J. Simonis, R. Ulrich, J. Wagner-Kuhr, S. Wayand, M. Weber, T. Weiler, S. Williamson, C. Wöhrmann, R. Wolf, G. Anagnostou, G. Daskalakis, T. Geralis, V. A. Giakoumopoulou, A. Kyriakis, D. Loukas, A. Psallidas, I. Topsis-Giotis, A. Agapitos, S. Kesisoglou, A. Panagiotou, N. Saoulidou, E. Tziaferi, I. Evangelou, G. Flouris, C. Foudas, P. Kokkas, N. Loukas, N. Manthos, I. Papadopoulos, E. Paradas, J. Strologas, G. Bencze, C. Hajdu, A. Hazi, P. Hidas, D. Horvath, F. Sikler, V. Veszpremi, G. Vesztergombi, A. J. Zsigmond, N. Beni, S. Czellar, J. Karancsi, J. Molnar, Z. Szillasi, M. Bartók, A. Makovec, P. Raics, Z. L. Trocsanyi, B. Ujvari, S. Choudhury, P. Mal, K. Mandal, D. K. Sahoo, N. Sahoo, S. K. Swain, S. Bansal, S. B. Beri, V. Bhatnagar, R. Chawla, R. Gupta, U. Bhawandeep, A. K. Kalsi, A. Kaur, M. Kaur, R. Kumar, A. Mehta, M. Mittal, J. B. Singh, G. Walia, Ashok Kumar, A. Bhardwaj, B. C. Choudhary, R. B. Garg, A. Kumar, S. Malhotra, M. Naimuddin, N. Nishu, K. Ranjan, R. Sharma, V. Sharma, R. Bhattacharya, S. Bhattacharya, K. Chatterjee, S. Dey, S. Dutta, S. Ghosh, N. Majumdar, A. Modak, K. Mondal, S. Mukhopadhyay, S. Nandan, A. Purohit, A. Roy, D. Roy, S. Roy Chowdhury, S. Sarkar, M. Sharan, R. Chudasama, D. Dutta, V. Jha, V. Kumar, A. K. Mohanty, L. M. Pant, P. Shukla, A. Topkar, T. Aziz, S. Banerjee, S. Bhowmik, R. M. Chatterjee, R. K. Dewanjee, S. Dugad, S. Ganguly, S. Ghosh, M. Guchait, A. Gurtu, Sa. Jain, G. Kole, S. Kumar, B. Mahakud, M. Maity, G. Majumder, K. Mazumdar, S. Mitra, G. B. Mohanty, B. Parida, T. Sarkar, N. Sur, B. Sutar, N. Wickramage, S. Chauhan, S. Dube, A. Kapoor, K. Kothekar, A. Rane, S. Sharma, H. Bakhshiansohi, H. Behnamian, S. M. Etesami, A. Fahim, M. Khakzad, M. Mohammadi Najafabadi, M. Naseri, S. Paktinat Mehdiabadi, F. Rezaei Hosseinabadi, B. Safarzadeh, M. Zeinali, M. Felcini, M. Grunewald, M. Abbrescia, C. Calabria, C. Caputo, A. Colaleo, D. Creanza, L. Cristella, N. De Filippis, M. De Palma, L. Fiore, G. Iaselli, G. Maggi, M. Maggi, G. Miniello, S. My, S. Nuzzo, A. Pompili, G. Pugliese, R. Radogna, A. Ranieri, G. Selvaggi, L. Silvestris, R. Venditti, G. Abbiendi, C. Battilana, D. Bonacorsi, S. Braibant-Giacomelli, L. Brigliadori, R. Campanini, P. Capiluppi, A. Castro, F. R. Cavallo, S. S. Chhibra, G. Codispoti, M. Cuffiani, G. M. Dallavalle, F. Fabbri, A. Fanfani, D. Fasanella, P. Giacomelli, C. Grandi, L. Guiducci, S. Marcellini, G. Masetti, A. Montanari, F. L. Navarria, A. Perrotta, A. M. Rossi, T. Rovelli, G. P. Siroli, N. Tosi, G. Cappello, M. Chiorboli, S. Costa, A. Di Mattia, F. Giordano, R. Potenza, A. Tricomi, C. Tuve, G. Barbagli, V. Ciulli, C. Civinini, R. D’Alessandro, E. Focardi, V. Gori, P. Lenzi, M. Meschini, S. Paoletti, G. Sguazzoni, L. Viliani, L. Benussi, S. Bianco, F. Fabbri, D. Piccolo, F. Primavera, V. Calvelli, F. Ferro, M. Lo Vetere, M. R. Monge, E. Robutti, S. Tosi, L. Brianza, M. E. Dinardo, S. Fiorendi, S. Gennai, R. Gerosa, A. Ghezzi, P. Govoni, S. Malvezzi, R. A. Manzoni, B. Marzocchi, D. Menasce, L. Moroni, M. Paganoni, D. Pedrini, S. Ragazzi, N. Redaelli, T. Tabarelli de Fatis, S. Buontempo, N. Cavallo, S. Di Guida, M. Esposito, F. Fabozzi, A. O. M. Iorio, G. Lanza, L. Lista, S. Meola, M. Merola, P. Paolucci, C. Sciacca, F. Thyssen, P. Azzi, N. Bacchetta, M. Bellato, L. Benato, D. Bisello, A. Boletti, R. Carlin, A. Carvalho Antunes De Oliveira, P. Checchia, M. Dall’Osso, T. Dorigo, U. Dosselli, F. Gasparini, U. Gasparini, A. Gozzelino, S. Lacaprara, M. Margoni, A. T. Meneguzzo, J. Pazzini, N. Pozzobon, P. Ronchese, F. Simonetto, E. Torassa, M. Tosi, S. Ventura, M. Zanetti, P. Zotto, A. Zucchetta, G. Zumerle, A. Braghieri, A. Magnani, P. Montagna, S. P. Ratti, V. Re, C. Riccardi, P. Salvini, I. Vai, P. Vitulo, L. Alunni Solestizi, G. M. Bilei, D. Ciangottini, L. Fanò, P. Lariccia, G. Mantovani, M. Menichelli, A. Saha, A. Santocchia, K. Androsov, P. Azzurri, G. Bagliesi, J. Bernardini, T. Boccali, R. Castaldi, M. A. Ciocci, R. Dell’Orso, S. Donato, G. Fedi, L. Foà, A. Giassi, M. T. Grippo, F. Ligabue, T. Lomtadze, L. Martini, A. Messineo, F. Palla, A. Rizzi, A. Savoy-Navarro, P. Spagnolo, R. Tenchini, G. Tonelli, A. Venturi, P. G. Verdini, L. Barone, F. Cavallari, G. D’imperio, D. Del Re, M. Diemoz, S. Gelli, C. Jorda, E. Longo, F. Margaroli, P. Meridiani, G. Organtini, R. Paramatti, F. Preiato, S. Rahatlou, C. Rovelli, F. Santanastasio, N. Amapane, R. Arcidiacono, S. Argiro, M. Arneodo, R. Bellan, C. Biino, N. Cartiglia, M. Costa, R. Covarelli, A. Degano, G. Dellacasa, N. Demaria, L. Finco, C. Mariotti, S. Maselli, E. Migliore, V. Monaco, E. Monteil, M. M. Obertino, L. Pacher, N. Pastrone, M. Pelliccioni, G. L. Pinna Angioni, F. Ravera, A. Romero, M. Ruspa, R. Sacchi, A. Solano, A. Staiano, S. Belforte, V. Candelise, M. Casarsa, F. Cossutti, G. Della Ricca, B. Gobbo, C. La Licata, A. Schizzi, A. Zanetti, A. Kropivnitskaya, S. K. Nam, D. H. Kim, G. N. Kim, M. S. Kim, D. J. Kong, S. Lee, Y. D. Oh, S. W. Lee, A. Sakharov, D. C. Son, J. A. Brochero Cifuentes, H. Kim, T. J. Kim, S. Song, S. Cho, S. Choi, Y. Go, D. Gyun, B. Hong, H. Kim, Y. Kim, B. Lee, K. Lee, K. S. Lee, S. Lee, J. Lim, S. K. Park, Y. Roh, H. D. Yoo, M. Choi, H. Kim, J. H. Kim, J. S. H. Lee, I. C. Park, G. Ryu, M. S. Ryu, Y. Choi, J. Goh, D. Kim, E. Kwon, J. Lee, I. Yu, V. Dudenas, A. Juodagalvis, J. Vaitkus, I. Ahmed, Z. A. Ibrahim, J. R. Komaragiri, M. A. B. Md Ali, F. Mohamad Idris, W. A. T. Wan Abdullah, M. N. Yusli, Z. Zolkapli, E. Casimiro Linares, H. Castilla-Valdez, E. De La Cruz-Burelo, I. Heredia-De La Cruz, A. Hernandez-Almada, R. Lopez-Fernandez, J. Mejia Guisao, A. Sanchez-Hernandez, S. Carrillo Moreno, F. Vazquez Valencia, I. Pedraza, H. A. Salazar Ibarguen, A. Morelos Pineda, D. Krofcheck, P. H. Butler, A. Ahmad, M. Ahmad, Q. Hassan, H. R. Hoorani, W. A. Khan, T. Khurshid, M. Shoaib, M. Waqas, H. Bialkowska, M. Bluj, B. Boimska, T. Frueboes, M. Górski, M. Kazana, K. Nawrocki, K. Romanowska-Rybinska, M. Szleper, P. Traczyk, P. Zalewski, G. Brona, K. Bunkowski, A. Byszuk, K. Doroba, A. Kalinowski, M. Konecki, J. Krolikowski, M. Misiura, M. Olszewski, M. Walczak, P. Bargassa, C. Beirão Da Cruz E Silva, A. Di Francesco, P. Faccioli, P. G. Ferreira Parracho, M. Gallinaro, J. Hollar, N. Leonardo, L. Lloret Iglesias, M. V. Nemallapudi, F. Nguyen, J. Rodrigues Antunes, J. Seixas, O. Toldaiev, D. Vadruccio, J. Varela, P. Vischia, S. Afanasiev, P. Bunin, M. Gavrilenko, I. Golutvin, I. Gorbunov, A. Kamenev, V. Karjavin, A. Lanev, A. Malakhov, V. Matveev, P. Moisenz, V. Palichik, V. Perelygin, S. Shmatov, S. Shulha, N. Skatchkov, V. Smirnov, A. Zarubin, V. Golovtsov, Y. Ivanov, V. Kim, E. Kuznetsova, P. Levchenko, V. Murzin, V. Oreshkin, I. Smirnov, V. Sulimov, L. Uvarov, S. Vavilov, A. Vorobyev, Yu. Andreev, A. Dermenev, S. Gninenko, N. Golubev, A. Karneyeu, M. Kirsanov, N. Krasnikov, A. Pashenkov, D. Tlisov, A. Toropin, V. Epshteyn, V. Gavrilov, N. Lychkovskaya, V. Popov, l. Pozdnyakov, G. Safronov, A. Spiridonov, E. Vlasov, A. Zhokin, R. Chistov, M. Danilov, O. Markin, V. Rusinov, E. Tarkovskii, V. Andreev, M. Azarkin, I. Dremin, M. Kirakosyan, A. Leonidov, G. Mesyats, S. V. Rusakov, A. Baskakov, A. Belyaev, E. Boos, M. Dubinin, L. Dudko, A. Ershov, A. Gribushin, V. Klyukhin, O. Kodolova, I. Lokhtin, I. Miagkov, S. Obraztsov, S. Petrushanko, V. Savrin, A Snigirev, I. Azhgirey, I. Bayshev, S. Bitioukov, V. Kachanov, A. Kalinin, D. Konstantinov, V. Krychkine, V. Petrov, R. Ryutin, A. Sobol, L. Tourtchanovitch, S. Troshin, N. Tyurin, A. Uzunian, A. Volkov, P. Adzic, P. Cirkovic, D. Devetak, J. Milosevic, V. Rekovic, J. Alcaraz Maestre, E. Calvo, M. Cerrada, M. Chamizo Llatas, N. Colino, B. De La Cruz, A. Delgado Peris, A. Escalante Del Valle, C. Fernandez Bedoya, J. P. Fernández Ramos, J. Flix, M. C. Fouz, P. Garcia-Abia, O. Gonzalez Lopez, S. Goy Lopez, J. M. Hernandez, M. I. Josa, E. Navarro De Martino, A. Pérez-Calero Yzquierdo, J. Puerta Pelayo, A. Quintario Olmeda, I. Redondo, L. Romero, M. S. Soares, J. F. de Trocóniz, M. Missiroli, D. Moran, J. Cuevas, J. Fernandez Menendez, S. Folgueras, I. Gonzalez Caballero, E. Palencia Cortezon, J. M. Vizan Garcia, I. J. Cabrillo, A. Calderon, J. R. Castiñeiras De Saa, E. Curras, P. De Castro Manzano, M. Fernandez, J. Garcia-Ferrero, G. Gomez, A. Lopez Virto, J. Marco, R. Marco, C. Martinez Rivero, F. Matorras, J. Piedra Gomez, T. Rodrigo, A. Y. Rodríguez-Marrero, A. Ruiz-Jimeno, L. Scodellaro, N. Trevisani, I. Vila, R. Vilar Cortabitarte, D. Abbaneo, E. Auffray, G. Auzinger, M. Bachtis, P. Baillon, A. H. Ball, D. Barney, A. Benaglia, L. Benhabib, G. M. Berruti, P. Bloch, A. Bocci, A. Bonato, C. Botta, H. Breuker, T. Camporesi, R. Castello, M. Cepeda, G. Cerminara, M. D’Alfonso, D. d’Enterria, A. Dabrowski, V. Daponte, A. David, M. De Gruttola, F. De Guio, A. De Roeck, E. Di Marco, M. Dobson, M. Dordevic, B. Dorney, T. du Pree, D. Duggan, M. Dünser, N. Dupont, A. Elliott-Peisert, G. Franzoni, J. Fulcher, W. Funk, D. Gigi, K. Gill, D. Giordano, M. Girone, F. Glege, R. Guida, S. Gundacker, M. Guthoff, J. Hammer, P. Harris, J. Hegeman, V. Innocente, P. Janot, H. Kirschenmann, V. Knünz, M. J. Kortelainen, K. Kousouris, P. Lecoq, C. Lourenço, M. T. Lucchini, N. Magini, L. Malgeri, M. Mannelli, A. Martelli, L. Masetti, F. Meijers, S. Mersi, E. Meschi, F. Moortgat, S. Morovic, M. Mulders, H. Neugebauer, S. Orfanelli, L. Orsini, L. Pape, E. Perez, M. Peruzzi, A. Petrilli, G. Petrucciani, A. Pfeiffer, M. Pierini, D. Piparo, A. Racz, T. Reis, G. Rolandi, M. Rovere, M. Ruan, H. Sakulin, J. B. Sauvan, C. Schäfer, C. Schwick, M. Seidel, A. Sharma, P. Silva, M. Simon, P. Sphicas, J. Steggemann, M. Stoye, Y. Takahashi, D. Treille, A. Triossi, A. Tsirou, G. I. Veres, N. Wardle, H. K. Wöhri, A. Zagozdzinska, W. D. Zeuner, W. Bertl, K. Deiters, W. Erdmann, R. Horisberger, Q. Ingram, H. C. Kaestli, D. Kotlinski, U. Langenegger, T. Rohe, F. Bachmair, L. Bäni, L. Bianchini, B. Casal, G. Dissertori, M. Dittmar, M. Donegà, P. Eller, C. Grab, C. Heidegger, D. Hits, J. Hoss, G. Kasieczka, P. Lecomte, W. Lustermann, B. Mangano, M. Marionneau, P. Martinez Ruiz del Arbol, M. Masciovecchio, M. T. Meinhard, D. Meister, F. Micheli, P. Musella, F. Nessi-Tedaldi, F. Pandolfi, J. Pata, F. Pauss, G. Perrin, L. Perrozzi, M. Quittnat, M. Rossini, M. Schönenberger, A. Starodumov, M. Takahashi, V. R. Tavolaro, K. Theofilatos, R. Wallny, T. K. Aarrestad, C. Amsler, L. Caminada, M. F. Canelli, V. Chiochia, A. De Cosa, C. Galloni, A. Hinzmann, T. Hreus, B. Kilminster, C. Lange, J. Ngadiuba, D. Pinna, G. Rauco, P. Robmann, D. Salerno, Y. Yang, K. H. Chen, T. H. Doan, Sh. Jain, R. Khurana, M. Konyushikhin, C. M. Kuo, W. Lin, Y. J. Lu, A. Pozdnyakov, S. S. Yu, Arun Kumar, P. Chang, Y. H. Chang, Y. W. Chang, Y. Chao, K. F. Chen, P. H. Chen, C. Dietz, F. Fiori, U. Grundler, W.-S. Hou, Y. Hsiung, Y. F. Liu, R.-S. Lu, M. Miñano Moya, E. Petrakou, J. F. Tsai, Y. M. Tzeng, B. Asavapibhop, K. Kovitanggoon, G. Singh, N. Srimanobhas, N. Suwonjandee, A. Adiguzel, M. N. Bakirci, S. Cerci, S. Damarseckin, Z. S. Demiroglu, C. Dozen, I. Dumanoglu, E. Eskut, S. Girgis, G. Gokbulut, Y. Guler, E. Gurpinar, I. Hos, E. E. Kangal, A. Kayis Topaksu, G. Onengut, K. Ozdemir, C. Zorbilmez, B. Bilin, S. Bilmis, B. Isildak, G. Karapinar, M. Yalvac, M. Zeyrek, E. Gülmez, M. Kaya, O. Kaya, E. A. Yetkin, T. Yetkin, A. Cakir, K. Cankocak, S. Sen, F. I. Vardarlı, B. Grynyov, L. Levchuk, P. Sorokin, R. Aggleton, F. Ball, L. Beck, J. J. Brooke, D. Burns, E. Clement, D. Cussans, H. Flacher, J. Goldstein, M. Grimes, G. P. Heath, H. F. Heath, J. Jacob, L. Kreczko, C. Lucas, Z. Meng, D. M. Newbold, S. Paramesvaran, A. Poll, T. Sakuma, S. Seif El Nasr-storey, S. Senkin, D. Smith, V. J. Smith, K. W. Bell, A. Belyaev, C. Brew, R. M. Brown, L. Calligaris, D. Cieri, D. J. A. Cockerill, J. A. Coughlan, K. Harder, S. Harper, E. Olaiya, D. Petyt, C. H. Shepherd-Themistocleous, A. Thea, I. R. Tomalin, T. Williams, S. D. Worm, M. Baber, R. Bainbridge, O. Buchmuller, A. Bundock, D. Burton, S. Casasso, M. Citron, D. Colling, L. Corpe, P. Dauncey, G. Davies, A. De Wit, M. Della Negra, P. Dunne, A. Elwood, D. Futyan, G. Hall, G. Iles, R. Lane, R. Lucas, L. Lyons, A.-M. Magnan, S. Malik, J. Nash, A. Nikitenko, J. Pela, B. Penning, M. Pesaresi, D. M. Raymond, A. Richards, A. Rose, C. Seez, A. Tapper, K. Uchida, M. Vazquez Acosta, T. Virdee, S. C. Zenz, J. E. Cole, P. R. Hobson, A. Khan, P. Kyberd, D. Leslie, I. D. Reid, P. Symonds, L. Teodorescu, M. Turner, A. Borzou, K. Call, J. Dittmann, K. Hatakeyama, H. Liu, N. Pastika, O. Charaf, S. I. Cooper, C. Henderson, P. Rumerio, D. Arcaro, A. Avetisyan, T. Bose, D. Gastler, D. Rankin, C. Richardson, J. Rohlf, L. Sulak, D. Zou, J. Alimena, G. Benelli, E. Berry, D. Cutts, A. Ferapontov, A. Garabedian, J. Hakala, U. Heintz, O. Jesus, E. Laird, G. Landsberg, Z. Mao, M. Narain, S. Piperov, S. Sagir, R. Syarif, R. Breedon, G. Breto, M. Calderon De La Barca Sanchez, S. Chauhan, M. Chertok, J. Conway, R. Conway, P. T. Cox, R. Erbacher, G. Funk, M. Gardner, W. Ko, R. Lander, C. Mclean, M. Mulhearn, D. Pellett, J. Pilot, F. Ricci-Tam, S. Shalhout, J. Smith, M. Squires, D. Stolp, M. Tripathi, S. Wilbur, R. Yohay, R. Cousins, P. Everaerts, A. Florent, J. Hauser, M. Ignatenko, D. Saltzberg, E. Takasugi, V. Valuev, M. Weber, K. Burt, R. Clare, J. Ellison, J. W. Gary, G. Hanson, J. Heilman, M. Ivova Paneva, P. Jandir, E. Kennedy, F. Lacroix, O. R. Long, M. Malberti, M. Olmedo Negrete, A. Shrinivas, H. Wei, S. Wimpenny, B. R. Yates, J. G. Branson, G. B. Cerati, S. Cittolin, R. T. D’Agnolo, M. Derdzinski, A. Holzner, R. Kelley, D. Klein, J. Letts, I. Macneill, D. Olivito, S. Padhi, M. Pieri, M. Sani, V. Sharma, S. Simon, M. Tadel, A. Vartak, S. Wasserbaech, C. Welke, F. Würthwein, A. Yagil, G. Zevi Della Porta, J. Bradmiller-Feld, C. Campagnari, A. Dishaw, V. Dutta, K. Flowers, M. Franco Sevilla, P. Geffert, C. George, F. Golf, L. Gouskos, J. Gran, J. Incandela, N. Mccoll, S. D. Mullin, J. Richman, D. Stuart, I. Suarez, C. West, J. Yoo, D. Anderson, A. Apresyan, J. Bendavid, A. Bornheim, J. Bunn, Y. Chen, J. Duarte, A. Mott, H. B. Newman, C. Pena, M. Spiropulu, J. R. Vlimant, S. Xie, R. Y. Zhu, M. B. Andrews, V. Azzolini, A. Calamba, B. Carlson, T. Ferguson, M. Paulini, J. Russ, M. Sun, H. Vogel, I. Vorobiev, J. P. Cumalat, W. T. Ford, A. Gaz, F. Jensen, A. Johnson, M. Krohn, T. Mulholland, U. Nauenberg, K. Stenson, S. R. Wagner, J. Alexander, A. Chatterjee, J. Chaves, J. Chu, S. Dittmer, N. Eggert, N. Mirman, G. Nicolas Kaufman, J. R. Patterson, A. Rinkevicius, A. Ryd, L. Skinnari, L. Soffi, W. Sun, S. M. Tan, W. D. Teo, J. Thom, J. Thompson, J. Tucker, Y. Weng, P. Wittich, S. Abdullin, M. Albrow, G. Apollinari, S. Banerjee, L. A. T. Bauerdick, A. Beretvas, J. Berryhill, P. C. Bhat, G. Bolla, K. Burkett, J. N. Butler, H. W. K. Cheung, F. Chlebana, S. Cihangir, V. D. Elvira, I. Fisk, J. Freeman, E. Gottschalk, L. Gray, D. Green, S. Grünendahl, O. Gutsche, J. Hanlon, D. Hare, R. M. Harris, S. Hasegawa, J. Hirschauer, Z. Hu, B. Jayatilaka, S. Jindariani, M. Johnson, U. Joshi, B. Klima, B. Kreis, S. Lammel, J. Lewis, J. Linacre, D. Lincoln, R. Lipton, T. Liu, R. Lopes De Sá, J. Lykken, K. Maeshima, J. M. Marraffino, S. Maruyama, D. Mason, P. McBride, P. Merkel, S. Mrenna, S. Nahn, C. Newman-Holmes, V. O’Dell, K. Pedro, O. Prokofyev, G. Rakness, E. Sexton-Kennedy, A. Soha, W. J. Spalding, L. Spiegel, S. Stoynev, N. Strobbe, L. Taylor, S. Tkaczyk, N. V. Tran, L. Uplegger, E. W. Vaandering, C. Vernieri, M. Verzocchi, R. Vidal, M. Wang, H. A. Weber, A. Whitbeck, D. Acosta, P. Avery, P. Bortignon, D. Bourilkov, A. Brinkerhoff, A. Carnes, M. Carver, D. Curry, S. Das, R. D. Field, I. K. Furic, J. Konigsberg, A. Korytov, K. Kotov, P. Ma, K. Matchev, H. Mei, P. Milenovic, G. Mitselmakher, D. Rank, R. Rossin, L. Shchutska, M. Snowball, D. Sperka, N. Terentyev, L. Thomas, J. Wang, S. Wang, J. Yelton, S. Linn, P. Markowitz, G. Martinez, J. L. Rodriguez, A. Ackert, J. R. Adams, T. Adams, A. Askew, S. Bein, J. Bochenek, B. Diamond, J. Haas, S. Hagopian, V. Hagopian, K. F. Johnson, A. Khatiwada, H. Prosper, M. Weinberg, M. M. Baarmand, V. Bhopatkar, S. Colafranceschi, M. Hohlmann, H. Kalakhety, D. Noonan, T. Roy, F. Yumiceva, M. R. Adams, L. Apanasevich, D. Berry, R. R. Betts, I. Bucinskaite, R. Cavanaugh, O. Evdokimov, L. Gauthier, C. E. Gerber, D. J. Hofman, P. Kurt, C. O’Brien, l. D. Sandoval Gonzalez, P. Turner, N. Varelas, Z. Wu, M. Zakaria, J. Zhang, B. Bilki, W. Clarida, K. Dilsiz, S. Durgut, R. P. Gandrajula, M. Haytmyradov, V. Khristenko, J.-P. Merlo, H. Mermerkaya, A. Mestvirishvili, A. Moeller, J. Nachtman, H. Ogul, Y. Onel, F. Ozok, A. Penzo, C. Snyder, E. Tiras, J. Wetzel, K. Yi, I. Anderson, B. A. Barnett, B. Blumenfeld, A. Cocoros, N. Eminizer, D. Fehling, L. Feng, A. V. Gritsan, P. Maksimovic, M. Osherson, J. Roskes, U. Sarica, M. Swartz, M. Xiao, Y. Xin, C. You, P. Baringer, A. Bean, C. Bruner, R. P. Kenny, D. Majumder, M. Malek, W. Mcbrayer, M. Murray, S. Sanders, R. Stringer, Q. Wang, A. Ivanov, K. Kaadze, S. Khalil, M. Makouski, Y. Maravin, A. Mohammadi, L. K. Saini, N. Skhirtladze, S. Toda, D. Lange, F. Rebassoo, D. Wright, C. Anelli, A. Baden, O. Baron, A. Belloni, B. Calvert, S. C. Eno, C. Ferraioli, J. A. Gomez, N. J. Hadley, S. Jabeen, R. G. Kellogg, T. Kolberg, J. Kunkle, Y. Lu, A. C. Mignerey, Y. H. Shin, A. Skuja, M. B. Tonjes, S. C. Tonwar, A. Apyan, R. Barbieri, A. Baty, R. Bi, K. Bierwagen, S. Brandt, W. Busza, I. A. Cali, Z. Demiragli, L. Di Matteo, G. Gomez Ceballos, M. Goncharov, D. Gulhan, Y. Iiyama, G. M. Innocenti, M. Klute, D. Kovalskyi, K. Krajczar, Y. S. Lai, Y.-J. Lee, A. Levin, P. D. Luckey, A. C. Marini, C. Mcginn, C. Mironov, S. Narayanan, X. Niu, C. Paus, C. Roland, G. Roland, J. Salfeld-Nebgen, G. S. F. Stephans, K. Sumorok, K. Tatar, M. Varma, D. Velicanu, J. Veverka, J. Wang, T. W. Wang, B. Wyslouch, M. Yang, V. Zhukova, A. C. Benvenuti, B. Dahmes, A. Evans, A. Finkel, A. Gude, P. Hansen, S. Kalafut, S. C. Kao, K. Klapoetke, Y. Kubota, Z. Lesko, J. Mans, S. Nourbakhsh, N. Ruckstuhl, R. Rusack, N. Tambe, J. Turkewitz, J. G. Acosta, S. Oliveros, E. Avdeeva, R. Bartek, K. Bloom, S. Bose, D. R. Claes, A. Dominguez, C. Fangmeier, R. Gonzalez Suarez, R. Kamalieddin, D. Knowlton, I. Kravchenko, F. Meier, J. Monroy, F. Ratnikov, J. E. Siado, G. R. Snow, B. Stieger, M. Alyari, J. Dolen, J. George, A. Godshalk, C. Harrington, I. Iashvili, J. Kaisen, A. Kharchilava, A. Kumar, S. Rappoccio, B. Roozbahani, G. Alverson, E. Barberis, D. Baumgartel, M. Chasco, A. Hortiangtham, A. Massironi, D. M. Morse, D. Nash, T. Orimoto, R. Teixeira De Lima, D. Trocino, R.-J. Wang, D. Wood, J. Zhang, S. Bhattacharya, K. A. Hahn, A. Kubik, J. F. Low, N. Mucia, N. Odell, B. Pollack, M. H. Schmitt, K. Sung, M. Trovato, M. Velasco, N. Dev, M. Hildreth, C. Jessop, D. J. Karmgard, N. Kellams, K. Lannon, N. Marinelli, F. Meng, C. Mueller, Y. Musienko, M. Planer, A. Reinsvold, R. Ruchti, N. Rupprecht, G. Smith, S. Taroni, N. Valls, M. Wayne, M. Wolf, A. Woodard, L. Antonelli, J. Brinson, B. Bylsma, L. S. Durkin, S. Flowers, A. Hart, C. Hill, R. Hughes, W. Ji, T. Y. Ling, B. Liu, W. Luo, D. Puigh, M. Rodenburg, B. L. Winer, H. W. Wulsin, O. Driga, P. Elmer, J. Hardenbrook, P. Hebda, S. A. Koay, P. Lujan, D. Marlow, T. Medvedeva, M. Mooney, J. Olsen, C. Palmer, P. Piroué, D. Stickland, C. Tully, A. Zuranski, S. Malik, A. Barker, V. E. Barnes, D. Benedetti, D. Bortoletto, L. Gutay, M. K. Jha, M. Jones, A. W. Jung, K. Jung, D. H. Miller, N. Neumeister, B. C. Radburn-Smith, X. Shi, I. Shipsey, D. Silvers, J. Sun, A. Svyatkovskiy, F. Wang, W. Xie, L. Xu, N. Parashar, J. Stupak, A. Adair, B. Akgun, Z. Chen, K. M. Ecklund, F. J. M. Geurts, M. Guilbaud, W. Li, B. Michlin, M. Northup, B. P. Padley, R. Redjimi, J. Roberts, J. Rorie, Z. Tu, J. Zabel, B. Betchart, A. Bodek, P. de Barbaro, R. Demina, Y. Eshaq, T. Ferbel, M. Galanti, A. Garcia-Bellido, J. Han, O. Hindrichs, A. Khukhunaishvili, K. H. Lo, P. Tan, M. Verzetti, J. P. Chou, E. Contreras-Campana, D. Ferencek, Y. Gershtein, E. Halkiadakis, M. Heindl, D. Hidas, E. Hughes, S. Kaplan, R. Kunnawalkam Elayavalli, A. Lath, K. Nash, H. Saka, S. Salur, S. Schnetzer, D. Sheffield, S. Somalwar, R. Stone, S. Thomas, P. Thomassen, M. Walker, M. Foerster, G. Riley, K. Rose, S. Spanier, K. Thapa, O. Bouhali, A. Castaneda Hernandez, A. Celik, M. Dalchenko, M. De Mattia, A. Delgado, S. Dildick, R. Eusebi, J. Gilmore, T. Huang, T. Kamon, V. Krutelyov, R. Mueller, I. Osipenkov, Y. Pakhotin, R. Patel, A. Perloff, D. Rathjens, A. Rose, A. Safonov, A. Tatarinov, K. A. Ulmer, N. Akchurin, C. Cowden, J. Damgov, C. Dragoiu, P. R. Dudero, J. Faulkner, S. Kunori, K. Lamichhane, S. W. Lee, T. Libeiro, S. Undleeb, I. Volobouev, E. Appelt, A. G. Delannoy, S. Greene, A. Gurrola, R. Janjam, W. Johns, C. Maguire, Y. Mao, A. Melo, H. Ni, P. Sheldon, S. Tuo, J. Velkovska, Q. Xu, M. W. Arenton, B. Cox, B. Francis, J. Goodell, R. Hirosky, A. Ledovskoy, H. Li, C. Neu, T. Sinthuprasith, X. Sun, Y. Wang, E. Wolfe, J. Wood, F. Xia, C. Clarke, R. Harr, P. E. Karchin, C. Kottachchi Kankanamge Don, P. Lamichhane, J. Sturdy, D. A. Belknap, D. Carlsmith, S. Dasu, L. Dodd, S. Duric, B. Gomber, M. Grothe, M. Herndon, A. Hervé, P. Klabbers, A. Lanaro, A. Levine, K. Long, R. Loveless, A. Mohapatra, I. Ojalvo, T. Perry, G. A. Pierro, G. Polese, T. Ruggles, T. Sarangi, A. Savin, A. Sharma, N. Smith, W. H. Smith, D. Taylor, P. Verwilligen, N. Woods, [Authorinst]The CMS Collaboration

**Affiliations:** 1 Yerevan Physics Institute, Yerevan, Armenia; 2 Institut für Hochenergiephysik der OeAW, Vienna, Austria; 3 National Centre for Particle and High Energy Physics, Minsk, Belarus; 4 Universiteit Antwerpen, Antwerp, Belgium; 5 Vrije Universiteit Brussel, Brussels, Belgium; 6 Université Libre de Bruxelles, Brussels, Belgium; 7 Ghent University, Ghent, Belgium; 8 Université Catholique de Louvain, Louvain-la-Neuve, Belgium; 9 Université de Mons, Mons, Belgium; 10 Centro Brasileiro de Pesquisas Fisicas, Rio de Janeiro, Brazil; 11 Universidade do Estado do Rio de Janeiro, Rio de Janeiro, Brazil; 12 Universidade Estadual Paulista, Universidade Federal do ABC, São Paulo, Brazil; 13 Institute for Nuclear Research and Nuclear Energy, Sofia, Bulgaria; 14 University of Sofia, Sofia, Bulgaria; 15 Beihang University, Beijing, China; 16 Institute of High Energy Physics, Beijing, China; 17 State Key Laboratory of Nuclear Physics and Technology, Peking University, Beijing, China; 18 Universidad de Los Andes, Bogotá, Colombia; 19 Faculty of Electrical Engineering, Mechanical Engineering and Naval Architecture, University of Split, Split, Croatia; 20 Faculty of Science, University of Split, Split, Croatia; 21 Institute Rudjer Boskovic, Zagreb, Croatia; 22 University of Cyprus, Nicosia, Cyprus; 23 Charles University, Prague, Czech Republic; 24 Academy of Scientific Research and Technology of the Arab Republic of Egypt, Egyptian Network of High Energy Physics, Cairo, Egypt; 25 National Institute of Chemical Physics and Biophysics, Tallinn, Estonia; 26 Department of Physics, University of Helsinki, Helsinki, Finland; 27 Helsinki Institute of Physics, Helsinki, Finland; 28 Lappeenranta University of Technology, Lappeenranta, Finland; 29 DSM/IRFU, CEA/Saclay, Gif-sur-Yvette, France; 30 Laboratoire Leprince-Ringuet, Ecole Polytechnique, IN2P3-CNRS, Palaiseau, France; 31 Institut Pluridisciplinaire Hubert Curien, Université de Strasbourg, Université de Haute Alsace Mulhouse, CNRS/IN2P3, Strasbourg, France; 32 Centre de Calcul de l’Institut National de Physique Nucleaire et de Physique des Particules, CNRS/IN2P3, Villeurbanne, France; 33 Institut de Physique Nucléaire de Lyon, Université de Lyon, Université Claude Bernard Lyon 1, CNRS-IN2P3, Villeurbanne, France; 34 Georgian Technical University, Tbilisi, Georgia; 35 Tbilisi State University, Tbilisi, Georgia; 36 I. Physikalisches Institut, RWTH Aachen University, Aachen, Germany; 37 III. Physikalisches Institut A, RWTH Aachen University, Aachen, Germany; 38 III. Physikalisches Institut B, RWTH Aachen University, Aachen, Germany; 39 Deutsches Elektronen-Synchrotron, Hamburg, Germany; 40 University of Hamburg, Hamburg, Germany; 41 Institut für Experimentelle Kernphysik, Karlsruhe, Germany; 42 Institute of Nuclear and Particle Physics (INPP), NCSR Demokritos, Aghia Paraskevi, Greece; 43 National and Kapodistrian University of Athens, Athens, Greece; 44 University of Ioánnina, Ioannina, Greece; 45 Wigner Research Centre for Physics, Budapest, Hungary; 46 Institute of Nuclear Research ATOMKI, Debrecen, Hungary; 47 University of Debrecen, Debrecen, Hungary; 48 National Institute of Science Education and Research, Bhubaneswar, India; 49 Panjab University, Chandigarh, India; 50 University of Delhi, Delhi, India; 51 Saha Institute of Nuclear Physics, Kolkata, India; 52 Bhabha Atomic Research Centre, Mumbai, India; 53 Tata Institute of Fundamental Research, Mumbai, India; 54 Indian Institute of Science Education and Research (IISER), Pune, India; 55 Institute for Research in Fundamental Sciences (IPM), Tehran, Iran; 56 University College Dublin, Dublin, Ireland; 57 INFN Sezione di Bari, Università di Bari, Politecnico di Bari, Bari, Italy; 58 INFN Sezione di Bologna, Università di Bologna, Bologna, Italy; 59 INFN Sezione di Catania, Università di Catania, Catania, Italy; 60 INFN Sezione di Firenze, Università di Firenze, Firenze, Italy; 61 INFN Laboratori Nazionali di Frascati, Frascati, Italy; 62 INFN Sezione di Genova, Università di Genova, Genova, Italy; 63 INFN Sezione di Milano-Bicocca, Università di Milano-Bicocca, Milan, Italy; 64 INFN Sezione di Napoli, Università di Napoli ‘Federico II’, Napoli, Italy, Università della Basilicata, Potenza, Italy, Università G. Marconi, Rome, Italy; 65 INFN Sezione di Padova, Università di Padova, Padova, Italy, Università di Trento, Trento, Italy; 66 INFN Sezione di Pavia, Università di Pavia, Pavia, Italy; 67 INFN Sezione di Perugia, Università di Perugia, Perugia, Italy; 68 INFN Sezione di Pisa, Università di Pisa, Scuola Normale Superiore di Pisa, Pisa, Italy; 69 INFN Sezione di Roma, Università di Roma, Rome, Italy; 70 INFN Sezione di Torino, Università di Torino, Turin, Italy, Università del Piemonte Orientale, Novara, Italy; 71 INFN Sezione di Trieste, Università di Trieste, Trieste, Italy; 72 Kangwon National University, Chuncheon, Korea; 73 Kyungpook National University, Daegu, Korea; 74 Chonbuk National University, Jeonju, Korea; 75 Institute for Universe and Elementary Particles, Chonnam National University, Kwangju, Korea; 76 Korea University, Seoul, Korea; 77 Seoul National University, Seoul, Korea; 78 University of Seoul, Seoul, Korea; 79 Sungkyunkwan University, Suwon, Korea; 80 Vilnius University, Vilnius, Lithuania; 81 National Centre for Particle Physics, Universiti Malaya, Kuala Lumpur, Malaysia; 82 Centro de Investigacion y de Estudios Avanzados del IPN, Mexico City, Mexico; 83 Universidad Iberoamericana, Mexico City, Mexico; 84 Benemerita Universidad Autonoma de Puebla, Puebla, Mexico; 85 Universidad Autónoma de San Luis Potosí, San Luis Potosí, Mexico; 86 University of Auckland, Auckland, New Zealand; 87 University of Canterbury, Christchurch, New Zealand; 88 National Centre for Physics, Quaid-I-Azam University, Islamabad, Pakistan; 89 National Centre for Nuclear Research, Swierk, Poland; 90 Institute of Experimental Physics, Faculty of Physics, University of Warsaw, Warsaw, Poland; 91 Laboratório de Instrumentação e Física Experimental de Partículas, Lisbon, Portugal; 92 Joint Institute for Nuclear Research, Dubna, Russia; 93 Petersburg Nuclear Physics Institute, Gatchina, St. Petersburg, Russia; 94 Institute for Nuclear Research, Moscow, Russia; 95 Institute for Theoretical and Experimental Physics, Moscow, Russia; 96 National Research Nuclear University ‘Moscow Engineering Physics Institute’ (MEPhI), Moscow, Russia; 97 P. N. Lebedev Physical Institute, Moscow, Russia; 98 Skobeltsyn Institute of Nuclear Physics, Lomonosov Moscow State University, Moscow, Russia; 99 State Research Center of Russian Federation, Institute for High Energy Physics, Protvino, Russia; 100 Faculty of Physics and Vinca Institute of Nuclear Sciences, University of Belgrade, Belgrade, Serbia; 101 Centro de Investigaciones Energéticas Medioambientales y Tecnológicas (CIEMAT), Madrid, Spain; 102 Universidad Autónoma de Madrid, Madrid, Spain; 103 Universidad de Oviedo, Oviedo, Spain; 104 Instituto de Física de Cantabria (IFCA), CSIC-Universidad de Cantabria, Santander, Spain; 105 CERN, European Organization for Nuclear Research, Geneva, Switzerland; 106 Paul Scherrer Institut, Villigen, Switzerland; 107 Institute for Particle Physics, ETH Zurich, Zurich, Switzerland; 108 Universität Zürich, Zurich, Switzerland; 109 National Central University, Chung-Li, Taiwan; 110 National Taiwan University (NTU), Taipei, Taiwan; 111 Department of Physics, Faculty of Science, Chulalongkorn University, Bangkok, Thailand; 112 Cukurova University, Adana, Turkey; 113 Physics Department, Middle East Technical University, Ankara, Turkey; 114 Bogazici University, Istanbul, Turkey; 115 Istanbul Technical University, Istanbul, Turkey; 116 Institute for Scintillation Materials of National Academy of Science of Ukraine, Kharkov, Ukraine; 117 National Scientific Center, Kharkov Institute of Physics and Technology, Kharkov, Ukraine; 118 University of Bristol, Bristol, UK; 119 Rutherford Appleton Laboratory, Didcot, UK; 120 Imperial College, London, UK; 121 Brunel University, Uxbridge, UK; 122 Baylor University, Waco, USA; 123 The University of Alabama, Tuscaloosa, USA; 124 Boston University, Boston, USA; 125 Brown University, Providence, USA; 126 University of California, Davis, Davis, USA; 127 University of California, Los Angeles, USA; 128 University of California, Riverside, Riverside, USA; 129 University of California, San Diego, La Jolla, USA; 130 University of California, Santa Barbara, Santa Barbara, USA; 131 California Institute of Technology, Pasadena, USA; 132 Carnegie Mellon University, Pittsburgh, USA; 133 University of Colorado Boulder, Boulder, USA; 134 Cornell University, Ithaca, USA; 135 Fermi National Accelerator Laboratory, Batavia, USA; 136 University of Florida, Gainesville, USA; 137 Florida International University, Miami, USA; 138 Florida State University, Tallahassee, USA; 139 Florida Institute of Technology, Melbourne, USA; 140 University of Illinois at Chicago (UIC), Chicago, USA; 141 The University of Iowa, Iowa City, USA; 142 Johns Hopkins University, Baltimore, USA; 143 The University of Kansas, Lawrence, USA; 144 Kansas State University, Manhattan, USA; 145 Lawrence Livermore National Laboratory, Livermore, USA; 146 University of Maryland, College Park, USA; 147 Massachusetts Institute of Technology, Cambridge, USA; 148 University of Minnesota, Minneapolis, USA; 149 University of Mississippi, Oxford, USA; 150 University of Nebraska-Lincoln, Lincoln, USA; 151 State University of New York at Buffalo, Buffalo, USA; 152 Northeastern University, Boston, USA; 153 Northwestern University, Evanston, USA; 154 University of Notre Dame, Notre Dame, USA; 155 The Ohio State University, Columbus, USA; 156 Princeton University, Princeton, USA; 157 University of Puerto Rico, Mayaguez, USA; 158 Purdue University, West Lafayette, USA; 159 Purdue University Calumet, Hammond, USA; 160 Rice University, Houston, USA; 161 University of Rochester, Rochester, USA; 162 Rutgers, The State University of New Jersey, Piscataway, USA; 163 University of Tennessee, Knoxville, USA; 164 Texas A&M University, College Station, USA; 165 Texas Tech University, Lubbock, USA; 166 Vanderbilt University, Nashville, USA; 167 University of Virginia, Charlottesville, USA; 168 Wayne State University, Detroit, USA; 169 University of Wisconsin-Madison, Madison, WI USA; 170 CERN, Geneva, Switzerland

## Abstract

A search is presented for narrow heavy resonances X decaying into pairs of Higgs bosons (${\mathrm{H}}$) in proton-proton collisions collected by the CMS experiment at the LHC at $\sqrt{s}=8\,\text {TeV}$. The data correspond to an integrated luminosity of 19.7$\,\text {fb}^{-1}$. The search considers ${\mathrm{H}} {\mathrm{H}}$ resonances with masses between 1 and 3$\,\text {TeV}$, having final states of two b quark pairs. Each Higgs boson is produced with large momentum, and the hadronization products of the pair of b quarks can usually be reconstructed as single large jets. The background from multijet and ${\mathrm{t}}\overline{{\mathrm{t}}}$ events is significantly reduced by applying requirements related to the flavor of the jet, its mass, and its substructure. The signal would be identified as a peak on top of the dijet invariant mass spectrum of the remaining background events. No evidence is observed for such a signal. Upper limits obtained at 95 % confidence level for the product of the production cross section and branching fraction $\sigma ({{\mathrm{g}} {\mathrm{g}}} \rightarrow \mathrm {X})\, \mathcal {B}({\mathrm {X}} \rightarrow {\mathrm{H}} {\mathrm{H}} \rightarrow {\mathrm{b}} \overline{{\mathrm{b}}} {\mathrm{b}} \overline{{\mathrm{b}}} )$ range from 10 to 1.5$\text {\,fb}$ for the mass of X from 1.15 to 2.0$\,\text {TeV}$, significantly extending previous searches. For a warped extra dimension theory with a mass scale $\Lambda _\mathrm {R} = 1$ $\,\text {TeV}$, the data exclude radion scalar masses between 1.15 and 1.55$\,\text {TeV}$.

## Introduction

The production of pairs of Higgs bosons (${\mathrm{H}}$) in the standard model (SM) has a predicted cross section in gluon–gluon fusion at $\sqrt{s}=8\,\text {TeV}$ [1, 2] for the Higgs boson mass $m_{{\mathrm{H}}} \approx 125\,\text {GeV}$ [3] of only $10.0 \pm 1.4\text {\,fb}$. Many BSM theories suggest the existence of narrow heavy particles $\mathrm {X}$   that can decay to a pair of Higgs bosons [4–12]. The natural width for such a resonance is expected to be a few percent of its pole mass $m_\mathrm {X}$, which corresponds to a typical detector resolution. In contrast, the SM production of Higgs boson pairs results in a broad distribution of effective mass, falling mainly in the range from 300 to 600$\,\text {GeV}$. Thus the presence of a narrow state would be readily detected, even if produced with a cross section as small as that for the SM process.

Searches for narrow particles decaying to two Higgs bosons have already been performed by the ATLAS [13–15] and CMS [16–19] collaborations in $\mathrm {p}$ $\mathrm {p}$ collisions at the CERN LHC. Until now their reach was limited to $m_\mathrm {X} \le 1.5\,\text {TeV}$. Because longitudinal $\mathrm {W}$  and ${\mathrm{Z}}$   states are provided by the Higgs field in the SM, any ${\mathrm{H}} {\mathrm{H}}$ resonance potentially also decays into $\mathrm {W}\mathrm {W}$ and ${\mathrm{Z}} {\mathrm{Z}}$ final states. Searches for $\mathrm {X} \rightarrow \mathrm {W}\mathrm {W}$, ${\mathrm{Z}} {\mathrm{Z}}$, and $\mathrm {W}{\mathrm{Z}}$ states were performed by ATLAS and CMS [20–24]. The combinations of these results [24–27] indicate that the region around $m_\mathrm {X} \approx 2\,\text {TeV}$ is particularly interesting to explore.

This paper reports on a search for $\mathrm {X} \rightarrow {\mathrm{H}} {\mathrm{H}}$ covering the mass range $1.15< m_\mathrm {X} < 3.0\,\text {TeV}$, significantly extending the reach of the present results beyond $1.5\,\text {TeV}$. The final state that provides the best sensitivity in this mass range is ${\mathrm{H}} {\mathrm{H}} \rightarrow {\mathrm{b}} \overline{{\mathrm{b}}} {\mathrm{b}} \overline{{\mathrm{b}}}$, which benefits from the expected large branching fraction ($\mathcal {B}$) of 57.7 % for ${\mathrm{H}} \rightarrow {\mathrm{b}} \overline{{\mathrm{b}}}$ [28] and a relatively low background from SM processes.

Many BSM proposals explicitly considered in this paper postulate the existence of a warped extra dimension (WED) [6] and predict the existence of a scalar radion [7–9]. The radion is a spin-0 resonance associated with the fluctuations in the length of the extra dimension. The production cross section as a function of $m_\mathrm {X}$ is proportional to $1/\Lambda _\mathrm {R} ^2$, where $\Lambda _\mathrm {R}$ is the scale parameter of the theory. In this paper we consider two cases: $\Lambda _\mathrm {R} = 1$ and $3\,\text {TeV}$. In the first case, the WED theory predicts a cross section that can be detected at the LHC [17], but is challenged by the constraints derived from the electroweak precision measurements [29]. This specific model is excluded up to $m_\mathrm {X} = 1.1\,\text {TeV}$ by the previous $\mathrm {X} \rightarrow {\mathrm{H}} {\mathrm{H}}$ searches [14, 17]. In contrast, the predicted cross section for $\Lambda _\mathrm {R} =3 \,\text {TeV}$ is a factor of 9 times smaller, but the theory is less constrained by these searches. We consider that the radion is produced exclusively via gluon-gluon fusion processes, with $\mathcal {B}(\text {radion} \rightarrow {\mathrm{H}} {\mathrm{H}}) \approx 25~\%$ above 1$\,\text {TeV}$.

In the mass range of this search, the topology of the ${\mathrm{b}} \overline{{\mathrm{b}}} {\mathrm{b}} \overline{{\mathrm{b}}}$ final state is constrained by the size of the Lorentz boost of the Higgs bosons that is typically $\gamma _{{\mathrm{H}}} \approx m_\mathrm {X}/ 2m_{{\mathrm{H}}} \gg 1$ and defines the so-called boosted regime [30–32]. In this regime each Higgs boson is produced with a large momentum and its decay products are collimated along its direction of motion. The hadronization of a pair of narrowly separated ${\mathrm{b}}$ quarks will result in a single reconstructed jet of mass compatible with $m_{{\mathrm{H}}}$. The ${\mathrm{H}}$ candidates are selected by employing jet substructure techniques to identify jets containing constituents with kinematics consistent with the decay of a highly boosted Higgs boson. These candidates are then required to be consistent with decays of ${\mathrm {B}}$ hadrons, based on our ${\mathrm{b}}$ tagging algorithms. The signal is identified in the dijet mass ($m_\mathrm {jj}$) spectrum as a peak above a falling background which originates mainly from multijet events and ${\mathrm{t}}\overline{{\mathrm{t}}}$ production.

## The CMS detector

The central feature of the CMS apparatus is a superconducting solenoid of 6$\text {\,m}$ internal diameter, providing a magnetic field of 3.8$\text {\,T}$. A silicon pixel and strip tracker, a lead tungstate crystal electromagnetic calorimeter, and a brass and scintillator hadron calorimeter, each composed of a barrel and two endcap sections, reside within the solenoid volume. Extensive forward calorimetry complements the coverage provided by the barrel and endcap detectors. Muons are measured in gas-ionization detectors embedded in the steel flux-return yoke outside the solenoid. A detailed description of the CMS detector, together with a definition of the coordinate system and the basic kinematic variables, can be found in Ref. [33].

## Simulated events

Monte Carlo (MC) simulations are used to provide: predictions of background processes, optimization of the event selection, and cross-checks of data-based background estimations.

Signal, multijet and ${\mathrm{t}}\overline{{\mathrm{t}}}$ background events are generated using the leading-order matrix element generator MadGraph 5v1.3.30 [34, 34]. Parton shower and hadronization are included using pythia 6.4.26 [35], and the matrix element is matched to the parton shower using the MLM scheme [36]. The Z2* tune is used to describe the underlying event. This tune is identical to the Z1 tune [37], but uses the CTEQ6L parton distribution functions (PDF) [38]. The signal events are simulated with an intrinsic width of the radion fixed to 1$\,\text {GeV}$, $m_{{\mathrm{H}}} =125\,\text {GeV}$. Different samples are generated for $m_\mathrm {X}$ ranging from 1.15 to 3 $\,\text {TeV}$. All generated events are processed through a simulation of the CMS apparatus based on Geant4  [39]. Additional $\mathrm {p}\mathrm {p}$ interactions within a bunch crossing (pileup) are added to the simulation, with a frequency distribution chosen to match that observed in data. During this data-taking period the mean number of interactions per bunch crossing is 21.

## Event reconstruction and selections

The analysis is based on data from $\mathrm {p}\mathrm {p}$ interactions observed with the CMS detector at $\sqrt{s}=8\,\text {TeV}$. The data correspond to an integrated luminosity of $19.7{\,\text {fb}^{-1}}$. Events are collected using at least one of the two specific trigger conditions based on jets reconstructed online: the first trigger requires a large $m_\mathrm {jj}$ calculated for the two jets of highest transverse momentum (referred to as leading jets); the second trigger requires a large value of $H_{\mathrm {T}} = \sum _i p_{\mathrm {T}} ^i$, where the sum runs over the reconstructed jets in the event with transverse momenta $p_{\mathrm {T}} >40\,\text {GeV}$. The lower thresholds applied to $m_\mathrm {jj}$ and the $H_{\mathrm {T}}$ triggers were changed during the data-taking period to maintain a constant trigger rate while the LHC peak luminosity steadily increased. More than half of the data were collected with $m_\mathrm {jj} > 750\,\text {GeV}$ and $H_{\mathrm {T}} > 650\,\text {GeV}$. The remaining data were collected with the requirement $H_{\mathrm {T}} >750\,\text {GeV}$.

Events are required to have at least one reconstructed $\mathrm {p}\mathrm {p}$ collision vertex within $|z | < 24\text {\,cm}$ of the center of the detector along the longitudinal beam directions. Many additional vertices, corresponding to pileup interactions, are usually reconstructed in an event using charged particle tracks. We assume that the primary interaction vertex corresponds to the one that maximizes the sum in $p_{\mathrm {T}} ^2$ of these associated tracks.

Individual particles are reconstructed using a particle-flow (PF) algorithm [40, 41] that combines the information from all the CMS detector components. Each such reconstructed particle is referred to as a PF candidate. The five classes of PF candidates correspond to muons, electrons, photons, and charged and neutral hadrons. Charged hadron candidates not originating from the primary vertex of the event are discarded to reduce contamination from pileup [42].

The Cambridge–Aachen (CA) algorithm [43], implemented in FastJet [44], clusters PF candidates into jets using a distance parameter $R = 0.8$. An event-by-event jet area-based correction [42, 45, 46] is applied to each reconstructed jet to remove the remaining energy originating from pileup vertices primarily consisting of neutral particles. The jet four-momenta are also corrected to account for the difference between the measured and the expected momentum at the particle level, using the standard CMS correction procedure described in Refs. [47, 48].

**Fig. 1:**
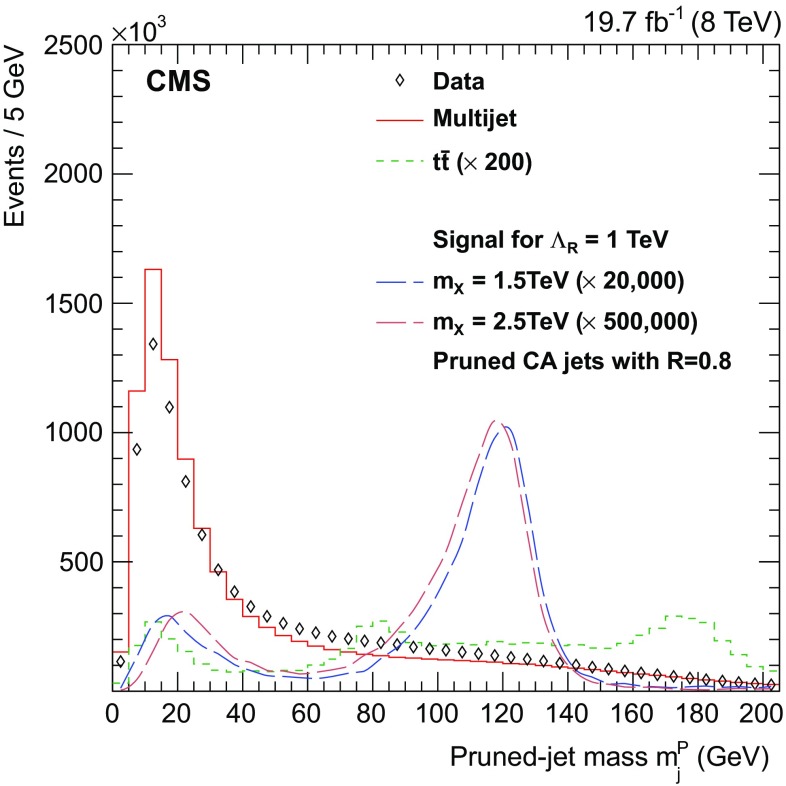
Simulated $m_\mathrm {j}^\mathrm {P}$ spectrum for spin-0 radion signals, multijet and ${\mathrm{t}}\overline{{\mathrm{t}}}$ events, and the spectrum measured in data. Each event contributes twice to the distribution, once per jet. The multijet contribution is rescaled to match the event yield in data, while the signal and ${\mathrm{t}}\overline{{\mathrm{t}}}$ spectra are rescaled by the large factors indicated, to be visible in the figure

Events are required to have at least two jets, and the two leading jets each to have $p_{\mathrm {T}} > 40\,\text {GeV}$ and pseudorapidity $|\eta | < 2.5$. In addition, identification criteria are applied to remove spurious jets associated with calorimeter noise [40]. To reduce the contribution from multijet events, the two leading jets must be relatively close in $\eta$, $|\Delta \eta _\mathrm {jj} | <1.3$, a selection discussed in Refs. [23, 49]. Events with $m_\mathrm {jj} <1\,\text {TeV}$ are rejected. Above this mass threshold, the efficiency of the trigger requirement for the chosen selections exceeds 99.5 %.

The mass and ${\mathrm{b}}$ flavour properties of the leading jets are used to suppress the multijet and ${\mathrm{t}}\overline{{\mathrm{t}}}$ backgrounds. Soft gluon radiation and a fraction of the remaining neutral pileup particles are first removed from each jet through the implementation of a jet-grooming algorithm called jet pruning [50, 51]. This technique reduces significantly the mass of jets originating from quarks and gluons [52], while improving the resolution of the jets resulting from the hadronic decays of a heavy SM boson [53]. The invariant mass $m_\mathrm {j}^\mathrm {P}$ is calculated for the two leading pruned jets. In Fig. 1, the $m_\mathrm {j}^\mathrm {P}$ distribution of the two leading jets is shown for data, signal, and background events. For jets initiated by a quark or a gluon, $m_\mathrm {j}^\mathrm {P}$ peaks around 15$\,\text {GeV}$, while jets from high-momentum Higgs boson decay usually have a pruned mass around 120$\,\text {GeV}$. The difference of $\approx$5$\,\text {GeV}$ relative to the nominal $m_{\mathrm{H}}$ value is related to the presence of neutrinos produced by the semileptonic decays of ${\mathrm {B}}$ mesons, and the inherent nature of the pruning procedure. A small peak near 15$\,\text {GeV}$ is also observed for signal events, and corresponds mainly to asymmetric decays in which the jet pruning algorithm removes the decay products of one of the two ${\mathrm {B}}$ mesons. Each of the leading jets has to satisfy $110<m_\mathrm {j}^\mathrm {P} <135\,\text {GeV}$, a requirement that is chosen to maximize the sensitivity of the analysis to the presence of a narrow resonances. Some differences are observed between the data and background estimated from simulation. These discrepancies do not affect the results of this analysis since the background is estimated using techniques based on data only.

The identification of jets likely to have originated from the hadronization of a pair of ${\mathrm{b}}$ quarks exploits the combined secondary vertex (CSV) ${\mathrm{b}}$ jet tagger [54]. This algorithm combines the information from track impact parameters and secondary vertices within a given jet into a continuous output discriminant  [54, 55]. The working point used in this paper corresponds to an efficiency of 80 % for identifying b jets and a rate of 10 % for mistagging jets from light quarks or gluons as originating from ${\mathrm{b}}$ quarks. This working point was chosen to maximize the sensitivity of the analysis, while retaining a sufficient number of events to allow a reliable estimation of the background.

In the first step of the procedure used to select ${\mathrm{H}}$ jet candidates, the pruned jets are split into two subjets by reversing the final iteration in the jet clustering algorithm. The angular separation between the subjets is $\Delta R \equiv \sqrt{{(\Delta \eta )^2 +(\Delta \phi )^2}}$, where $\eta$ is the pseudorapidity and $\phi$ the azimuthal angle. Two cases are considered, with the transition between them occurring at $m_\mathrm {X} \approx 1.6\,\text {TeV}$:

1. $\Delta R>0.3$: in this group the jet is considered to be ${\mathrm{b}}$ tagged if at least one subjet satisfies the requirements of the CSV working point. Moreover, the jet is considered as “double ${\mathrm{b}}$ tagged” if both subjets satisfy the CSV requirement.
2. $\Delta R<0.3$: here the subjet ${\mathrm{b}}$ tagging selection is inefficient [55]. The ${\mathrm{b}}$ tagging algorithm is therefore applied directly to the jet. In this case it is not possible to distinguish between ${\mathrm{b}}$-tagged and double ${\mathrm{b}}$-tagged jets, and therefore either of these two possibilities are accepted.

In summary, a jet is considered an ${\mathrm{H}}$ jet candidate if it satisfies the mass and ${\mathrm{b}}$ tagging requirements. Events are selected when both leading jets are ${\mathrm{H}}$ jets, and at least one of them is double ${\mathrm{b}}$ tagged. The simulated results are corrected to match the ${\mathrm{H}}$ and ${\mathrm{b}}$ tagging efficiencies observed in data [55].

**Table 1:** Summary of selection requirements, with their signal and ${\mathrm{t}}\overline{{\mathrm{t}}}$ background efficiencies, and total number of events observed in data. The selection criteria are applied sequentially and the efficiencies are cumulative, except in the last section of the table dedicated to categorization

Selection criteria	Efficiency for				Observed events (%)
	Signal with $m_\mathrm {X}$ ($\text {TeV}$)			${\mathrm{t}}\overline{{\mathrm{t}}}$ (%)	
	1.3 (%)	2.0 (%)	3.0 (%)		
Fiducial acceptance					
At least 2 jets with $p_{\mathrm {T}} >40\,\text {GeV}$,					
$|\eta | <2.5$, and $|\Delta \eta _\mathrm {jj} | < 1.3$	63	61	59	29	
Analysis selections					
$m_\mathrm {jj} > 1\,\text {TeV}$	59	59	58	3.5	2 677 308
2 jets with $110<m_\mathrm {j}^\mathrm {P} <135\,\text {GeV}$	12	12	8.5	0.29	9 977
2 ${\mathrm{b}}$-tagged jets and					
$\ge$1 double ${\mathrm{b}}$ tagged jets	9.0	8.5	4.5	0.05	217
2 jets with $\tau _{21} < 0.75$ and					
$\ge$1 jet with $\tau _{21} < 0.5$	8.6	8.1	4.0	0.04	162
Categorization					
HPHP	6.3	5.5	2.4	0.03	63
HPLP	1.1	1.2	0.9	0.007	48
LPHP	1.2	1.4	0.7	0.004	51

A final selection is based on the kinematic properties of the constituents of ${\mathrm{H}}$ jets. The quantity N-subjettiness [56–58] $\tau _N$ is used to quantify the degree to which constituents of a jet can be arranged into *N* subjets. The ratio $\tau _{21} = \tau _2/\tau _1$ is calculated for each of the two ${\mathrm{H}}$ jet candidates. High- (HP) and low-purity (LP) Higgs boson candidates are defined as having $\tau _{21} < 0.5$ and $0.5 \le \tau _{21} < 0.75$, respectively. Events are required to have at least one HP ${\mathrm{H}}$ jet and another ${\mathrm{H}}$ jet that passes either the HP or LP requirements.

The sample of events satisfying the previously defined criteria is subsequently divided into three categories. Events with two high-purity ${\mathrm{H}}$ jets form the HPHP category. Among the remaining events, those for which the high-purity ${\mathrm{H}}$ jet is the leading jet constitute the HPLP category. The rest of the sample constitutes the LPHP category.

The selection criteria applied to reduce the background are summarized in Table 1. The region of phase space defined by all these criteria is referred to as the signal region. The fraction of the simulated signal and ${\mathrm{t}}\overline{{\mathrm{t}}}$ samples, satisfying these criteria, as well as the number of data events passing the selections is also provided.

**Fig. 2:**
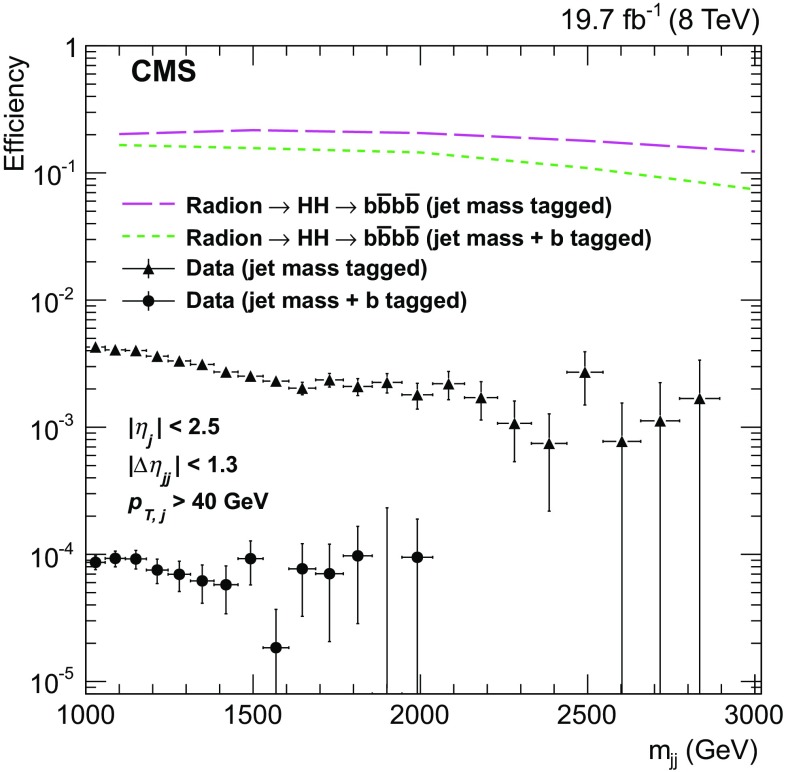
The efficiencies of ${\mathrm{H}}$ mass requirement and combined ${\mathrm{H}}$ mass and ${\mathrm{b}}$ tagging criteria, for data and signal. Events are required to be in the fiducial region ($|\eta | < 2.5$, $p_{\mathrm {T}} >40\,\text {GeV}$ for both jets and $|\Delta \eta _\mathrm {jj} | <1.3$). The horizontal bar on each data point indicates the width of the bin

The fiducial selection is defined by the two leading jets having $|\eta | < 2.5$, $p_{\mathrm {T}} >40\,\text {GeV}$, and a separation $|\Delta \eta _\mathrm {jj} | <1.3$. The fraction of the signal within this fiducial region depends on its spin, and is $\approx$60 % for a spin-0 resonance. The efficiency of the combined ${\mathrm{H}}$ mass and ${\mathrm{b}}$ tagging criteria for events within the fiducial region, for signal and data, is shown in Fig. 2. The number of data events is reduced by four orders of magnitude while the signal efficiencies range from 10 to 20 % with a weak dependence on $m_\mathrm {X}$, and are observed to be independent of the spin of the resonance. Finally, the total acceptance times efficiency is provided in Table 1, and varies between 4.0 and 8.8 %, with the largest fraction of events populating the HPHP category.

Figure 2 shows that the probability of incorrectly identifying multijet or ${\mathrm{t}}\overline{{\mathrm{t}}}$ events as events with two Higgs bosons is less than 0.1 %, and appears to be independent of $m_\mathrm {jj}$ within statistical uncertainties. A more precise quantification is provided in Table 1 for ${\mathrm{t}}\overline{{\mathrm{t}}}$ events. In particular, we observe that the dijet mass, the pruned jet mass, and ${\mathrm{b}}$ tagging criteria are each sufficient for reducing the ${\mathrm{t}}\overline{{\mathrm{t}}}$ background by an order of magnitude. In contrast, the N-subjettiness criterion is inefficient in reducing it.

## Signal extraction

The signal is identified in the binned $m_\mathrm {jj}$ spectrum in bin widths chosen to match the resolution of the dijet mass, as described in Ref. [59]. This resolution is $\approx$50$\,\text {GeV}$ at $m_\mathrm {X} = 1.15\,\text {TeV}$, increasing slowly to $\approx$100$\,\text {GeV}$ for $m_\mathrm {X} = 3\,\text {TeV}$.

The analysis defines a likelihood, for each $m_\mathrm {X}$ hypothesis, based on the total number of events in data, signal, and background counted in a mass window in each category. These mass windows have a typical size of three or four bins centered approximatively around $m_\mathrm {X}$ (see Table 2) and contains more than 95 % of signal events. The amount of signal is estimated in the mass window using MC simulation, while the amount of background is estimated as the integral of a parameterized model. The total likelihood combines the information from the three event categories.

**Table 2:** Mass windows used for different signal hypotheses

$m_\mathrm {X}$	Mass window	$m_\mathrm {X}$	Mass window
($\text {GeV}$ )	($\text {GeV}$ )	($\text {GeV}$ )	($\text {GeV}$ )
1150	[1058, 1246]	1700	[1607, 1856]
1200	[1118, 1313]	1800	[1687, 1945]
1300	[1181, 1455]	1900	[1700, 2037]
1400	[1313, 1530]	2000	[1856, 2132]
1500	[1383, 1607]	2500	[2231, 2775]
1600	[1455, 1770]	3000	[2775, 3279]

## Parameterization of background

After event selection, $\approx$75, 90, and 95 % of the total background is expected to originate from multijet events in HPHP, HPLP, and LPHP categories, respectively. The remaining contribution is from ${\mathrm{t}}\overline{{\mathrm{t}}}$ production, which is modelled in simulation, and rescaled to the total next-to-next-to-leading order cross section [60]. All other backgrounds containing Higgs bosons or $\mathrm {W}$/${\mathrm{Z}}$ bosons decaying into jets represent less than 1 % of the total background.

The total background is estimated from data, without separating the multijet or ${\mathrm{t}}\overline{{\mathrm{t}}}$ fractions. The expected $m_\mathrm {jj}$ background spectrum is approximated by a falling exponential for $1< m_\mathrm {jj} < 3\,\text {TeV}$,

1 $$\begin{aligned} \frac{{\mathrm{d}}N_\text {Background}}{{\mathrm{d}}m_\mathrm {jj}} = N_B\, a\, {\mathrm{e}}^{-a(m_\mathrm {jj}-1000\,\text {GeV})}, \end{aligned}$$

where the parameterization has been chosen to minimize the correlation between the normalization $N_B$ and slope *a*. We obtain *a* from a fit to the $m_\mathrm {jj}$ distribution in a control region, defined as the portion of phase space where one of the jets satisfies $110< m_\mathrm {j}^\mathrm {P} < 135\,\text {GeV}$ and the other jet is required to have $60< m_\mathrm {j}^\mathrm {P} < 100\,\text {GeV}$. This choice of the window for $m_\mathrm {j}^\mathrm {P}$ results from a compromise between limited signal contamination, sufficiently large statistics, and similarity in substructure properties between the sideband jet and the ${\mathrm{H}}$ jet. To use this control region we assume that there is no resonant signal in the Z${\mathrm{H}}$ final state.

**Fig. 3:**
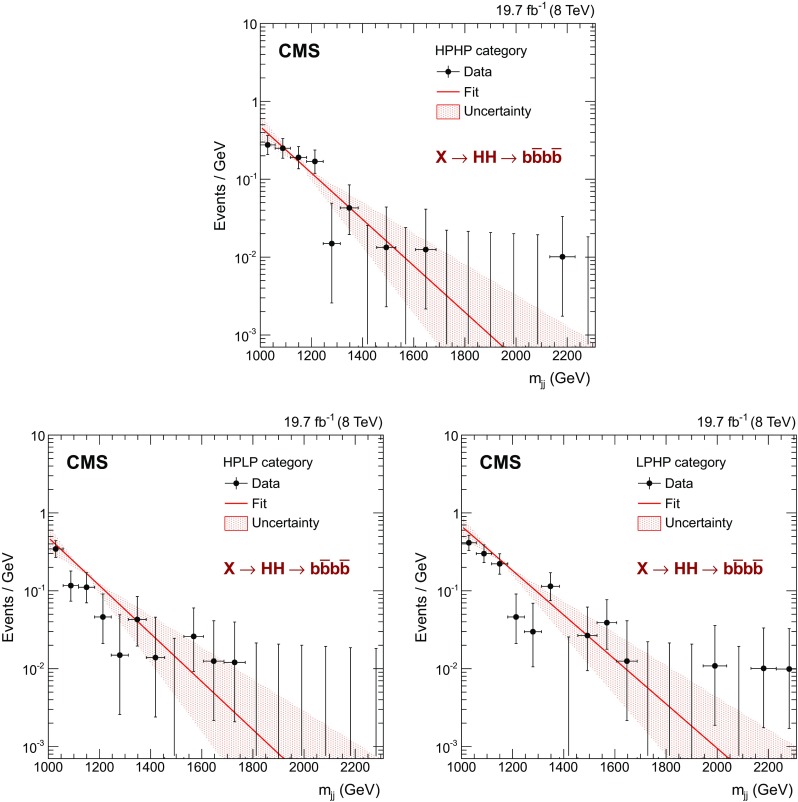
Observed $m_\mathrm {jj}$ spectrum (*black points*) in the control region together with the superimposed background fit (*red line*) and the uncertainty associated with the variation of the slope parameter *a* (*red shaded area*) for HPHP (*top*), HPLP (*bottom-left*), and LPHP (*bottom-right*) categories

The control region contains between 1.1–2 times the number of events in the signal region depending on the category. The result of the fit and the uncertainty band associated with the uncertainty in the parameter *a* are shown in Fig. 3. The effect of a residual contamination of the control region by the signal is explicitly checked by adding an ${\mathrm{H}} {\mathrm{H}}$ signal to the control region at different masses, with a typical $\sigma ({\mathrm{g}} {\mathrm{g}} \rightarrow \mathrm {X} \rightarrow {\mathrm{H}} {\mathrm{H}}) \, \mathcal {B}(\mathrm {X} \rightarrow {\mathrm{H}} {\mathrm{H}} \rightarrow {\mathrm{b}} \overline{{\mathrm{b}}} {\mathrm{b}} \overline{{\mathrm{b}}} )$, corresponding to the sensitivity of the analysis at a given $m_\mathrm {X}$. The change in the slope parameter *a* is observed to be negligible.

We extract $N_B$ for each signal hypothesis from the fit to the data that excludes events in the counting window described in Sect. 5. This background extraction procedure motivates the choice of the lower value of the $m_\mathrm {X}$ window for which the search is performed. In order to improve the constraint on $N_B$, there must be at least one bin on the left side of the mass window to be retained.

This background estimation procedure assumes, on the one hand, that the $m_\mathrm {jj}$ spectrum is similar in the signal and the control regions, and on the other hand, that it is similar for multijet and ${\mathrm{t}}\overline{{\mathrm{t}}}$ event samples. The following cross-checks are performed to validate these hypotheses:

- The similarity of distributions for the signal and control regions are confirmed in the simulated multijet sample.
- The parameters *a* and $N_B$ are extracted from the signal region (using an approach similar to that of Ref. [23]), and found to be compatible within statistical uncertainties with the parameters obtained through the normal method of background estimation.
- The bin-by-bin normalization between the signal and control regions is calculated using a sideband obtained by inverting the ${\mathrm{b}}$ tagging criterion on one of the jets (using a technique similar to that in Ref. [61]), and the normalization factor found to be independent of $m_\mathrm {jj}$, within the statistical uncertainties.
- The ${\mathrm{t}}\overline{{\mathrm{t}}}$ contribution in the signal region obtained from simulation is fitted by the function in Eq. (1) and the resulting fit is found to be consistent with the distribution of the overall background within the statistical uncertainties.

Closure checks of the background-estimation procedure are performed using simulated multijet events. These are also performed directly in data in the control region. For this purpose, the control region is split in two, a low mass control region with $60<m_\mathrm {j}^\mathrm {P} <90\,\text {GeV}$, and a pseudo-signal region with $90<m_\mathrm {j}^\mathrm {P} <100\,\text {GeV}$. In both cases, the predicted background is found to be compatible with that observed, within the statistical uncertainties.

## Systematic uncertainties

The largest contributions to the systematic uncertainty in the signal yields are the uncertainties associated with the classification of the events into the purity categories, the estimation of the efficiency to identify a ${\mathrm{H}}$ jet, and the calculation of the total integrated luminosity (2.6 %) [62], as well as with the determination of the jet energy scale (JES) and resolution (JER). The major systematic uncertainties are summarized in Table 3.

**Table 3:** Typical uncertainties in different categories

Source	Uncertainty
Background (statistical)	15 – 100 %
Signal (systematic)	
Luminosity	2.6 %
${\mathrm{b}}$ tagging	3.8–14.4 %
Mass tagging	$5.2 \oplus 3.0~\%$
JES $\oplus$ JER	$1.0 \oplus 1.0~\%$
Categorization	$^{+25}_{-19}~\%$ (HPHP), $^{+59}_{-37}~\%$ (HPLP), $^{+59}_{-37}~\%$ (LPHP)

**Fig. 4:**
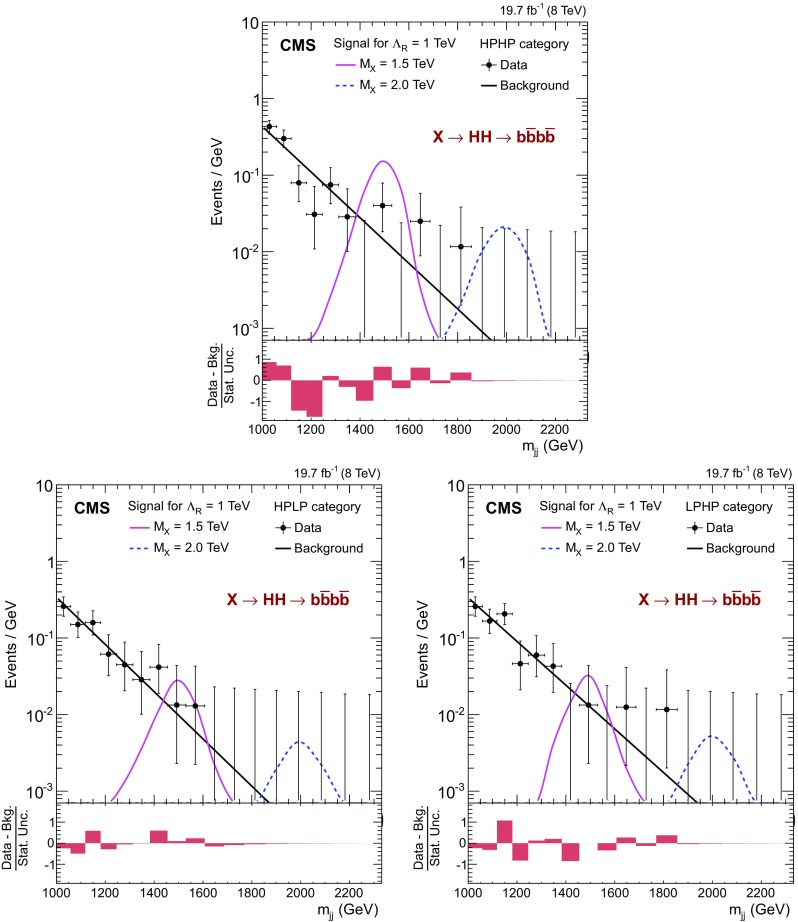
Observed $m_\mathrm {jj}$ spectrum (*black points*) compared with a background estimate (*black line*), obtained in background only hypothesis, for HPHP (*top*), HPLP (*bottom-left*), and LPHP (*bottom-right*) categories. The simulated radion resonances of $m_\mathrm {X} = 1.5$ and 2$\,\text {TeV}$ are also shown. The *lower panel* in each plot shows the difference between the number of observed and estimated background events divided by the statistical uncertainty estimated from data

**Fig. 5:**
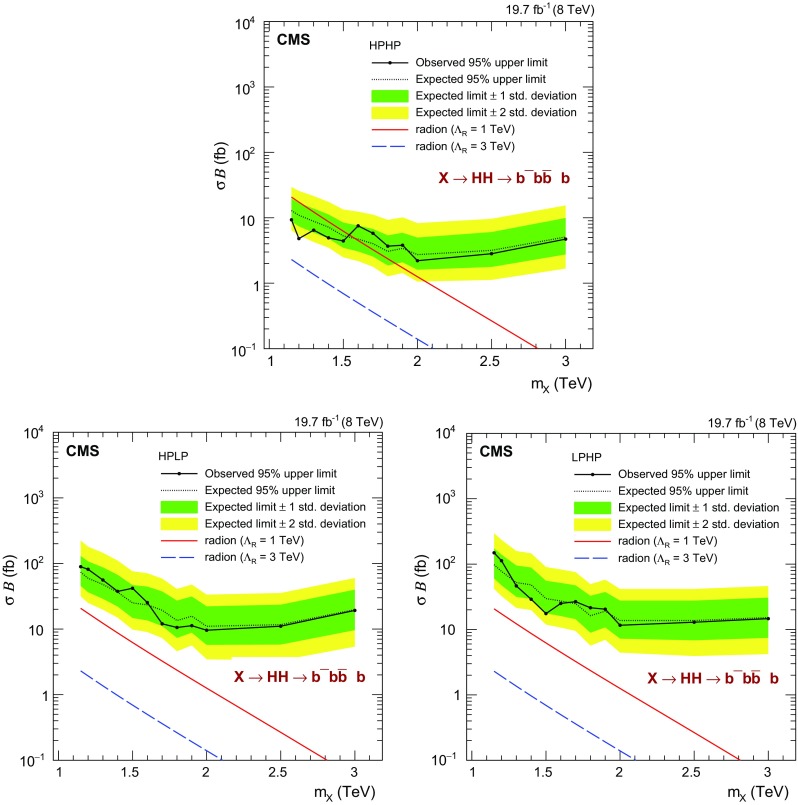
Observed and expected 95 % CL *upper limits* on the product of cross section of a narrow resonance and the branching fraction $\sigma ({\mathrm{g}} {\mathrm{g}} \rightarrow \mathrm {X}) \, \mathcal {B}(\mathrm {X} \rightarrow {\mathrm{H}} {\mathrm{H}} \rightarrow {\mathrm{b}} \overline{{\mathrm{b}}} {\mathrm{b}} \overline{{\mathrm{b}}} )$. Theory *curves* corresponding to WED models with radion are superimposed

The uncertainty in the ${\mathrm{b}}$ tagging efficiency originates from the uncertainty in the data-to-simulation scale factors that are applied to the simulated signal [55]. The scale factors are $\approx$90 % with an absolute uncertainty between ±3.8 % and ±14 %, depending on the value of $m_\mathrm {X}$. The uncertainty increases at large $m_\mathrm {X}$ because of the limited amount of data available to constrain the scale factors.

The uncertainty in the mass selection efficiency is 2.6 % for each jet and 5.2 % for the event. This uncertainty is estimated by studying high $p_{\mathrm {T}}$ $\mathrm {W}$ bosons in a ${\mathrm{t}}\overline{{\mathrm{t}}}$ data control sample [53] and comparing to MC predictions. It includes the effect of the difference in fragmentation between light and ${\mathrm{b}}$ quarks. This uncertainty is fully correlated for all ${\mathrm{H}}$ jets. In addition, the impact of the pileup modelling uncertainty in the Higgs boson mass-tagging efficiency is assumed to be 1.5 % per jet, i.e., 3 % for the event [23].

An uncertainty accounting for possible migration of signal events from the HPHP to the HPLP and LPHP categories results in uncertainties of $+25$ and $-19~\%$, and of $+59$ and $-37~\%$ in the normalization of the HPHP category, and of both the HPLP and LPHP categories, respectively. These uncertainties are estimated by comparing the $\tau _{21}$ distribution in measured and simulated ${\mathrm{t}}\overline{{\mathrm{t}}}$ events  [23, 53]. It also includes a quantification of the difference between the fragmentation of $\mathrm {W}$ and Higgs bosons decaying hadronically. The fraction of signal events that do not enter any of the three categories changes from 2 % at 1.1$\,\text {TeV}$ to 20 % at 3.0$\,\text {TeV}$. The uncertainty associated with migration out of the three categories is estimated to be much smaller than that associated with migration within them.

The uncertainties in the JES (1–2 %) [48] and JER (10 %) [47] impact the signal acceptance in the $m_\mathrm {jj}$ counting window. Each of these systematic contributions provide less than 1 % uncertainty in the normalization of the expected signal events.

In summary, the uncertainty in the signal normalization associated with the migration of signal events between categories is larger than the total contribution of all other uncertainties, which varies from 7 % at $m_\mathrm {X} = 1.1\,\text {TeV}$ to 15 % at $m_\mathrm {X} = 3\,\text {TeV}$.

The statistical uncertainty in the total background ranges from 15 % at 1.3$\,\text {TeV}$ up to 100 % at 3$\,\text {TeV}$. It is calculated by generating pseudo-experiments in the signal and control regions, assuming Poisson fluctuations in the number of events in each bin about its central value. For low $m_\mathrm {jj}$, the statistical precision is limited by the uncertainty in the parameter $N_B$, and for high masses, by the uncertainty in the slope parameter *a*. The impact of the choice of the functional form used in the parameterization of the background distribution is evaluated by comparing the results from the exponential fit to those from an alternative power-law function, and is found to be negligible compared to the statistical uncertainty.

The uncertainty related to the efficiency of the $\tau _{21}$ tagger is assumed to be fully correlated between the HPLP and LPHP categories and anticorrelated with the HPHP category. The uncertainties in the background estimate are uncorrelated between categories, while all other uncertainties are expected to be fully correlated among all three categories.

## Results

The observed data are shown separately for the three event categories in Fig. 4. For comparison, we also show the predictions obtained for the background-only hypothesis. The $N_B$ normalization parameter is extracted for all events in the signal region with $1< m_\mathrm {jj} < 3\,\text {TeV}$. The bottom panel of each plot shows the difference between the observed data and the predicted background, divided by the statistical uncertainty estimated in the data. The background model describes the data within their statistical uncertainties. The events with the largest masses in the HPHP, HPLP, and LPHP categories are at $m_\mathrm {jj} = 1780$, 1560, and 1800$\,\text {GeV}$, respectively.

**Fig. 6:**
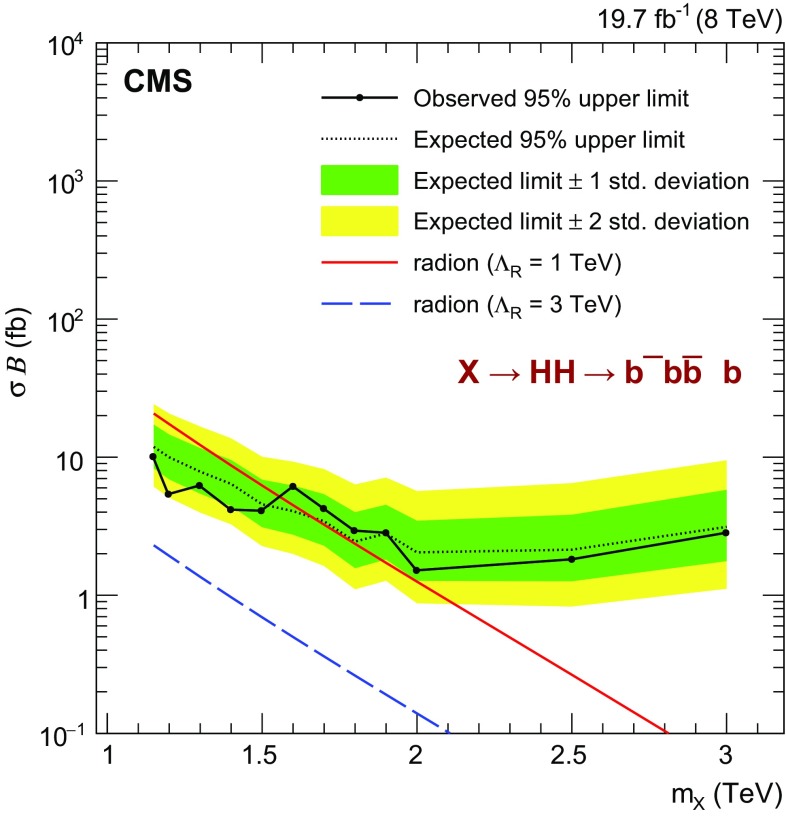
Observed and expected 95 % CL *upper limits* on the product of cross section of a narrow resonance and the branching fraction $\sigma ({\mathrm{g}} {\mathrm{g}} \rightarrow \mathrm {X}) \, \mathcal {B}(\mathrm {X} \rightarrow {\mathrm{H}} {\mathrm{H}} \rightarrow {\mathrm{b}} \overline{{\mathrm{b}}} {\mathrm{b}} \overline{{\mathrm{b}}} )$. Theory *curves* corresponding to WED models with radion are also shown

Upper limits on the cross section for the production of resonances are extracted using the asymptotic approximation of the CL$_\mathrm {s}$ method [63, 64]. Figure 5 shows the observed and expected 95 % confidence level (CL) upper limits on the product of the cross section and the branching fraction $\sigma ({\mathrm{g}} {\mathrm{g}} \rightarrow \mathrm {X}) \, \mathcal {B}(\mathrm {X} \rightarrow {\mathrm{H}} {\mathrm{H}} \rightarrow {\mathrm{b}} \overline{{\mathrm{b}}} {\mathrm{b}} \overline{{\mathrm{b}}} )$ obtained for each event category. The HPHP category is always the most sensitive, nevertheless above 2$\,\text {TeV}$ the HPLP and LPHP categories are also important because of inefficiencies in N-subjettiness at high $p_{\mathrm {T}}$. Figure 6 and Table 4 provide the combined limits. The excluded cross sections at 95 % CL vary from 10$\text {\,fb}$ at 1.15$\,\text {TeV}$ to 1.5$\text {\,fb}$ at 2$\,\text {TeV}$. Above 2$\,\text {TeV}$ the excluded cross sections increase to 2.8$\text {\,fb}$ at 3$\,\text {TeV}$, since the sensitivity is limited by the increasing inefficiency of ${\mathrm{H}}$ jet identification, as described in Sect. 4.

**Fig. 7:**
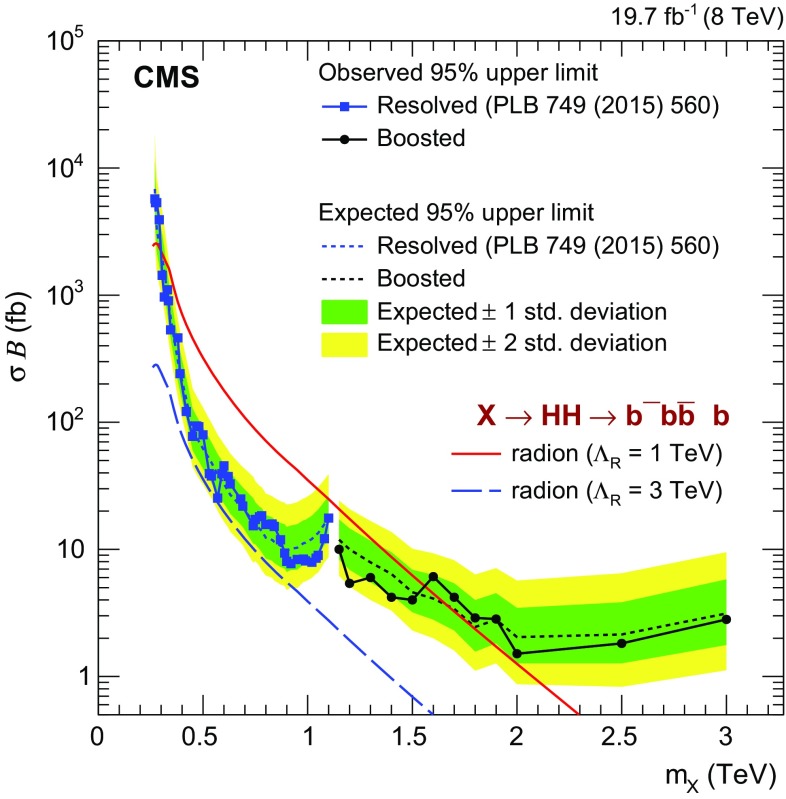
Observed and expected 95 % CL upper limits on the product of cross section of a narrow resonance and the branching fraction $\sigma ({\mathrm{g}} {\mathrm{g}} \rightarrow \mathrm {X}) \, \mathcal {B}(\mathrm {X} \rightarrow {\mathrm{H}} {\mathrm{H}} \rightarrow {\mathrm{b}} \overline{{\mathrm{b}}} {\mathrm{b}} \overline{{\mathrm{b}}} )$. Theory predictions corresponding to WED models with a radion are also shown. Results from the resolved analysis of Ref. [17] are shown by *blue squares*. For clarity, only a representative subset of the points are provided from the resolved analysis. The result from this paper is shown in *black dots*

Figure 7 extends the $\mathrm {X} \rightarrow {\mathrm{H}} {\mathrm{H}} \rightarrow {\mathrm{b}} \overline{{\mathrm{b}}} {\mathrm{b}} \overline{{\mathrm{b}}}$ search down to $m_\mathrm {X} = 260\,\text {GeV}$ by including limits from Ref. [17]. This search, referred to as the resolved analysis, considers a case where the decay products from two Higgs bosons are reconstructed as four jets. It is interesting to observe that the sensitivity of the resolved analysis starts to degrade at $m_\mathrm {X} \approx 1\,\text {TeV}$. At this point the typical angular distance between two jets from one Higgs boson reaches $\Lambda _{R} = 4m_{{\mathrm{H}}}/m_\mathrm {X} \approx 0.5$ and the two jets overlap [30]. Above 1.1$\,\text {TeV}$ the boosted analysis becomes more sensitive.

**Table 4:** Observed and expected 95 % CL upper limits on the product of cross section and the branching fraction $\sigma ({\mathrm{g}} {\mathrm{g}} \rightarrow \mathrm {X}) \, \mathcal {B}(\mathrm {X} \rightarrow {\mathrm{H}} {\mathrm{H}} \rightarrow {\mathrm{b}} \overline{{\mathrm{b}}} {\mathrm{b}} \overline{{\mathrm{b}}} )$ for HPHP, HPLP and LPHP categories combined. The one standard deviation on the 95 % CL upper limit is also provided

$m_\mathrm {X}$	Observed limit	Expected limit ${\pm }1\sigma$
($$\text {GeV}$$ )	(fb)	(fb)
1150	10.0	11.9$\; \pm ^{5.3}_{3.6}$
1200	5.4	10.0$\; \pm ^{4.6}_{3.1}$
1300	6.0	7.9$\; \pm ^{3.8}_{2.4}$
1400	4.2	6.4$\; \pm ^{3.1}_{2.0}$
1500	4.0	4.6$\; \pm ^{2.3}_{1.4}$
1600	6.1	4.1$\; \pm ^{2.2}_{1.3}$
1700	4.2	3.4$\; \pm ^{2.0}_{1.1}$
1800	2.9	2.5$\; \pm ^{1.5}_{0.9}$
1900	2.8	2.8$\; \pm ^{1.7}_{1.0}$
2000	1.5	2.0$\; \pm ^{1.4}_{0.9}$
2500	1.8	2.1$\; \pm ^{1.7}_{0.9}$
3000	2.8	3.1$\; \pm ^{2.7}_{1.4}$

To quantify the sensitivity of this analysis to new physics, the limits are compared to predictions of radion production for $\Lambda _\mathrm {R} = 1$ and $3\,\text {TeV}$, as shown in Fig. 6. We find that a radion corresponding to $\Lambda _\mathrm {R} = 1\,\text {TeV}$ is excluded by the boosted analysis alone, for masses between 1.15 and 1.55$\,\text {TeV}$. This result extends the limits already set by the resolved analysis from 0.3 to 1.1 $\,\text {TeV}$.

## Summary

A search is presented for narrow heavy resonances decaying into a pair of Higgs bosons in proton-proton collisions collected by the CMS experiment at $\sqrt{s}=8\,\text {TeV}$. The full data sample of $19.7{\,\text {fb}^{-1}}$is explored. The background from multijet and ${\mathrm{t}}\overline{{\mathrm{t}}}$ events is significantly reduced by applying requirements related to the flavor of the jet, its mass, and its substructure. No significant excess of events is observed above the background expected from the SM processes. The results are interpreted as exclusion limits at 95 % confidence on the production cross section for $m_\mathrm {X}$ between 1.15 and 3.0$\,\text {TeV}$, extending significantly beyond 1.5 TeV the reach of previous searches. A radion with scale parameter $\Lambda _\mathrm {R} = 1\,\text {TeV}$ decaying into ${\mathrm{H}} {\mathrm{H}}$ is excluded for $1.15< m_\mathrm {X} <1.55\,\text {TeV}$ for the first time in direct searches.
